# Crystal structure of *cis*-7,8-dihy­droxy-5,10,15,20-tetra­phenyl­chlorin and its zinc(II)–ethyl­enedi­amine complex

**DOI:** 10.1107/S2056989022002729

**Published:** 2022-03-15

**Authors:** Nivedita Chaudhri, Christian Brückner, Matthias Zeller

**Affiliations:** a Department of Chemistry, University of Connecticut, Storrs, CT 06269-3060, USA; b Department of Chemistry, Purdue University, 560 Oval Drive, West Lafayette, IN, 47907-2084, USA

**Keywords:** crystal structure, porphyrinoids, hydro­porphyrins, *meso*-phenyl­chlorins, β-hy­droxy­chlorin

## Abstract

The title chlorin, **2^Ph^H_2_**, hydrogen-bonded to di­methyl­amino­pyridine (DMAP), C_44_H_32_N_4_O_2_·C_7_H_10_N_2_, and its corresponding zinc(II) complex, **2^Ph^Zn**, axially coordinated to ethyl­enedi­amine (EDA), [Zn(C_44_H_30_N_4_O_2_)]·C_2_H_8_N_2_, were isolated and crystallized by adventitious reduction of the corresponding osmate esters by DMAP and EDA, respectively. Known since 1996 and, *inter alia*, used for the preparation of a wide range of (planar and non-planar) chlorin analogues (so-called pyrrole-modified porphyrins), their conformational analyses in the solid state are important benchmarks. Both macrocycles are only modestly distorted from planarity and both are slightly more non-planar than the corresponding dimeth­oxy-derivative, but less planar than a free-base *meso*-penta­fluoro­phenyl-based osmate ester. NSD analyses provide qu­anti­tative and qualitative analyses of the distortion modes. One origin of the non-planarity is presumably the avoidance of the eclipsed configuration of the two *vic–cis* diols on the pyrroline moiety; the resulting deformation of the pyrroline translates in some cases into the macrocycle. The structure of **2^Ph^H_2_** features voids making up *ca* 26% of the unit-cell volume filled with highly disordered solvate mol­ecules (chloro­form and hexa­nes). **2^Ph^Zn** crystallized with a 13.6 (4)% occupied solvate methanol mol­ecule.

## Abstract 2

Normal mode structural decomposition (NSD) shows the title chlorin compounds to have considerable saddling deformation from planarity.

## Chemical context

The study of synthetic chlorins as functional, spectroscopic, or structural models for nature’s premiere light-harvesting pigment chloro­phyll is one of the central aspects in contemporary porphyrinoid chemistry (Flitsch, 1988 ▸; Liu *et al.*, 2018 ▸; Taniguchi & Lindsey, 2017 ▸; Lindsey, 2015 ▸). Because of the facility of the synthesis of a wide range of *meso*-tetra­aryl­porphyrins, their conversion to chlorins has been widely studied (Flitsch, 1988 ▸; Taniguchi & Lindsey, 2017 ▸).

**Figure 1:**
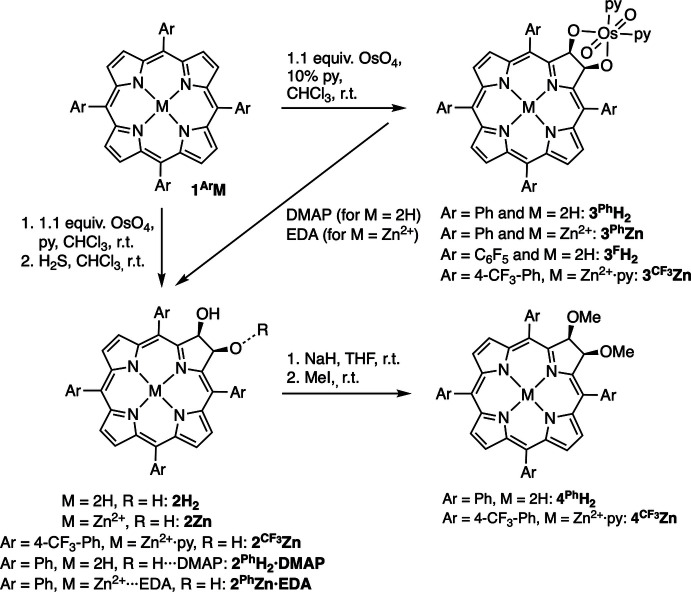
Synthetic pathways towards **2^Ph^H_2_·DMAP** and **2^Ph^Zn·EDA** and their meth­oxy ethers.

We contributed to the field the description of the OsO_4_-mediated di­hydroxy­lation of *meso*-tetra­aryl­porphyrins **1^Ar^M**, generating the corresponding chlorin diols **2^Ar^M** (Fig. 1 ▸) (Brückner & Dolphin, 1995*a* ▸; Brückner *et al.*, 1998 ▸). Depending on the stoichiometric ratio of OsO_4_ used and whether the porphyrin metal complex or free base is used, the reaction may also lead to the regioselective formation of tetra­hydroxy­metalloisobacteriochlorins or tetra­hydroxy­bacteriochlorins, respectively (Brückner & Dolphin, 1995*b* ▸; Samankumara *et al.*, 2010 ▸; Hyland *et al.*, 2012 ▸; Bruhn & Brückner, 2015 ▸). Chlorin diols **2^Ar^H_2_** have shown efficacy as photosensitizers in photodynamic therapy (Macalpine *et al.*, 2002 ▸) or are substrates toward their oxidation to the corres­ponding diones (Starnes *et al.*, 2000 ▸, 2001 ▸; Daniell *et al.*, 2003 ▸). Importantly, chlorin diols **2^Ar^M** are the starting materials for the generation of a wide range of planar and non-planar chlorin analogues (so-called pyrrole-modified porphyrins) (Brückner, 2016 ▸; Sharma *et al.*, 2017 ▸; Hewage *et al.*, 2019 ▸; Brückner *et al.*, 2020 ▸; Luciano *et al.*, 2020 ▸; Wu *et al.*, 2020 ▸), whereby the parent chlorin diols **2^Ph^H_2_** and **2^Ph^Zn** generally serve as spectroscopic benchmarks. Since the conformation of a porphyrinic macrocycle greatly influences its electronic structure, the structural characterization of the benchmark compounds **2^Ph^H_2_** and **2^Ph^Zn** is important. Curiously, however, even though these fundamental compounds are known since 1996, crystals suitable for single X-ray crystal structure analyses could not be grown to date. However, related derivatives, such as osmate ester **3^F^H_2_** (Hewage *et al.*, 2019 ▸), a number of tetra­hydroxy­bacteriochlorins and isobacteriochlorins (Samankumara *et al.*, 2010 ▸), and a number of alkyl­ated diol free base and metal complexes **4^Ar^** ***M*** (*M* = 2H, Ni, Cu, Zn, Pd) (Samankumara *et al.*, 2010 ▸; Sharma *et al.*, 2017 ▸) could be structurally characterized. 
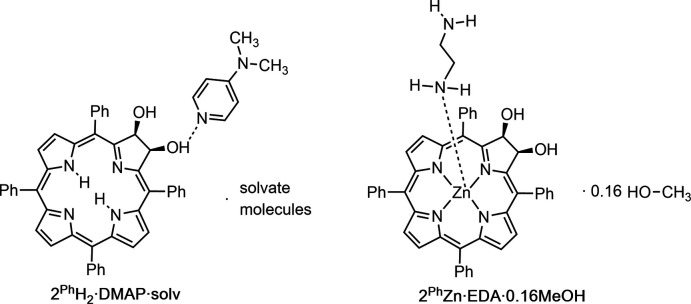


In due course of working with the inter­mediate osmate esters and attempts to form crystals of the amine adducts, we inadvertently reduced the osmate ester and the long-sought parent free base *meso*-phenyl chlorin diol **2^Ph^H_2_**, as **2^Ph^H_2_·DMAP** hydrogen-bonded to DMAP (4-di­methyl­amino­pyridine) and the zinc(II) complex **2^Ph^Zn**, in the form **2^Ph^Zn·EDA** in which the metal is axially coordinated to ethyl­enedi­amine (EDA), crystallized in single-crystal X-ray diffraction quality.

## Structural commentary

**Figure 2:**
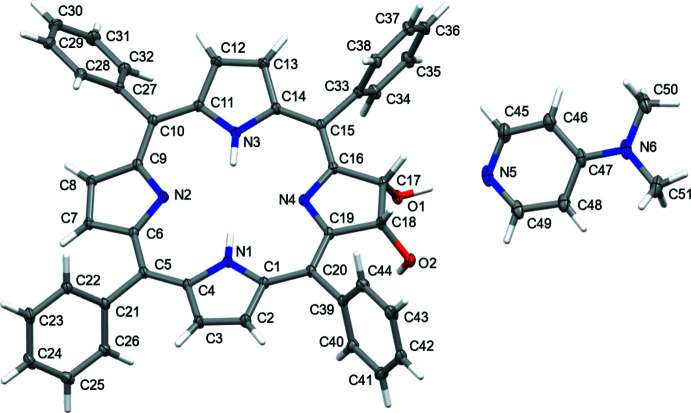
X-ray structure of **2^Ph^H_2_·DMAP** with the atom-labeling scheme for non-H atoms. 50% probability ellipsoids.

The structures of both **2^Ph^H_2_·DMAP** and **2^Ph^Zn·EDA** confirm the *cis–vic* stereochemistry of the diol functionality and the near-perpendicular arrangement of the *meso*-phenyl groups – structural features well known for these types of *meso*-aryl­chlorin diols (Hewage *et al.*, 2019 ▸; Samankumara *et al.*, 2010 ▸; Sharma *et al.*, 2017 ▸) or *meso*-aryl­porphyrinoids, in general (Senge, 2000 ▸) (Figs. 2 ▸ and 3 ▸).

**Figure 4:**
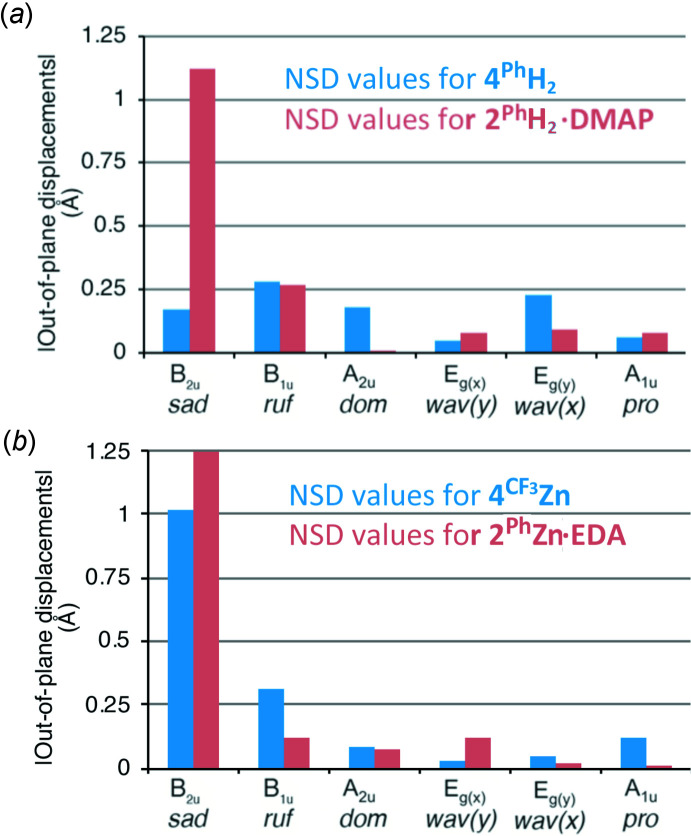
Normal mode Structural Decomposition (NSD) analysis (Kingsbury & Senge, 2021 ▸) of (*a*), the chromophore conformations of di­hydroxy­chlorin **2^Ph^H_2_·DMAP** (hydrogen-bonded to DMAP) in comparison to the conformation of the chromophore of di­meth­oxy­chlorin **4^Ph^H_2_** (Samankumara *et al.*, 2010 ▸), and (*b*), the equivalent chromophore conformation analysis of **2^Ph^Zn·EDA** in comparison to the closely related dimeth­oxy derivative **4^CF3^Zn** (Sharma *et al.*, 2017 ▸).

Importantly, the structures allow the determination of the conformation of their chromophores. The dissection of the conformation of **2^Ph^H_2_·DMAP** using a normal mode structural decomposition (NSD) analysis (Kingsbury & Senge, 2021 ▸; Shelnutt *et al.*, 1998 ▸) shows that its chromophore exhibits a considerable saddling distortion. In comparison, the dimeth­oxy derivative **4^Ph^H_2_** (Samankumara *et al.*, 2010 ▸) is more planar, with only very modest distortions evenly spread over a number of distortion modes (Fig. 4 ▸ *a*). In **4^Ph^H_2_**, both meth­oxy substituents point toward the outside, whereas the corresponding hy­droxy groups in **2^Ph^H_2_·DMAP** point in opposite directions, with only the hydrogen-bonded (to DMAP) hy­droxy group pointing outwards. A slight deformation of the pyrroline moiety in **2^Ph^H_2_·DMAP** alleviates the steric inter­actions between the two hy­droxy groups [26.65 (13)° O—C—C—O torsion angle] that would be otherwise forced to be eclipsed. The corresponding torsion angle in **4^Ph^H_2_** is slightly smaller [17.23 (17)°; Samankumara *et al.*, 2010 ▸]. This *vic-*-*cis*-substituents-induced pyrroline deformation was also observed previously (Sharma *et al.*, 2017 ▸; Hewage *et al.*, 2019 ▸).

**Figure 5:**
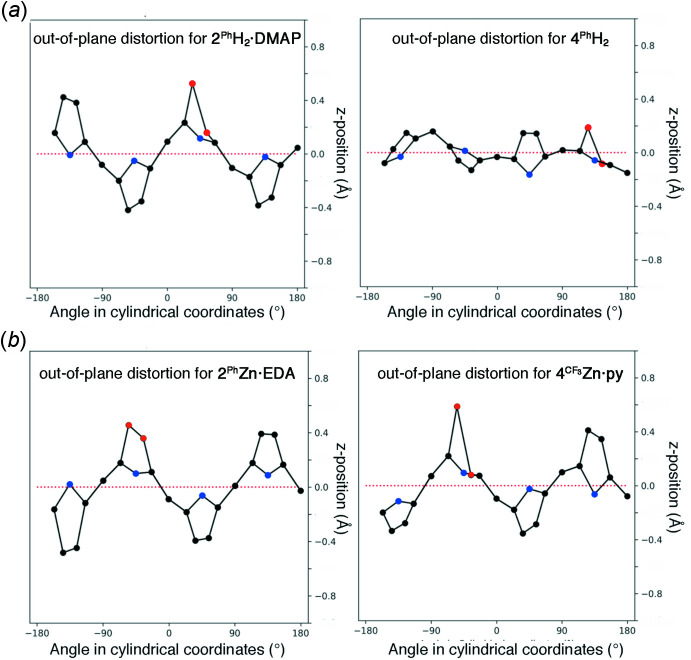
Out-of-plane plots (Kingsbury & Senge, 2021 ▸) of the chromophore conformations of (*a*), di­hydroxy­chlorin **2^Ph^H_2_·DMAP** and di­meth­oxy­chlorin **4^Ph^H_2_** (Samankumara *et al.*, 2010 ▸), and (*b*), the equivalent plots of **2^Ph^Zn·EDA** and **4^CF3^Zn·py** (Sharma *et al.*, 2017 ▸). The atoms indicated in red are the pyrroline β-carbons carrying the *cis*-hy­droxy or meth­oxy groups.

The out-of-plane plots (Kingsbury & Senge, 2021 ▸) of the two free-base chlorins **2^Ph^H_2_·DMAP** and **4^Ph^H_2_** also illustrate the qualitative and qu­anti­tative differences in the conformations of the two (Fig. 5 ▸ *a*).

The saddling deformation is more pronounced in the corresponding zinc(II) complexes but the deformation modes observed in either of the complexes are very similar (Fig. 4 ▸ *b* and 5*b*). This (small) *B*_2*u*_ deformation mode is typical for penta-coordinated, square-pyramidal porphyrinoid zinc(II) complexes (Kingsbury & Senge, 2021 ▸). The differences in conformation quality and qu­antity is only minimal between the parent compound **2^Ph^Zn·EDA** and its *p*-aryl-substituted and methyl­ated analogue **4^CF3^Zn·py**. In addition, both mol­ecules carry their axial ligand on the same hemisphere defined by the macrocycle the diol/dimeth­oxy moieties are located. Nonetheless, there are differences. For instance, a smaller O—C—C—O torsion angle was observed in the diol zinc complex **2^Ph^Zn·EDA** [O—C_β_—C_β_—O dihedral angle = 7.86 (17)°], whereas the corresponding angle in the dimeth­oxy derivative **4^CF3^Zn** is 28.1 (4)°(Sharma *et al.*, 2017 ▸).

In neither the free base nor the zinc complex of the diol chlorins are any significant in-plane deformations observed. The change in the macrocycle conformation upon methyl­ation and/or hydrogen bonding to an amine acceptor reiterates the conformational malleability of the chlorin chromophore (Kratky *et al.*, 1985 ▸), as previously also shown in the varying conformations of a range of transition-metal complexes (Sharma *et al.*, 2017 ▸).

## Supra­molecular features

The dominant supra­molecular inter­actions in both **2^Ph^H_2_·DMAP** and **2^Ph^Zn·EDA** are hydrogen-bonding inter­actions between the hydroxyl functions of the chlorin mol­ecules, and the DMAP and EDA bases incorporated into the crystal structure.

**Figure 6:**
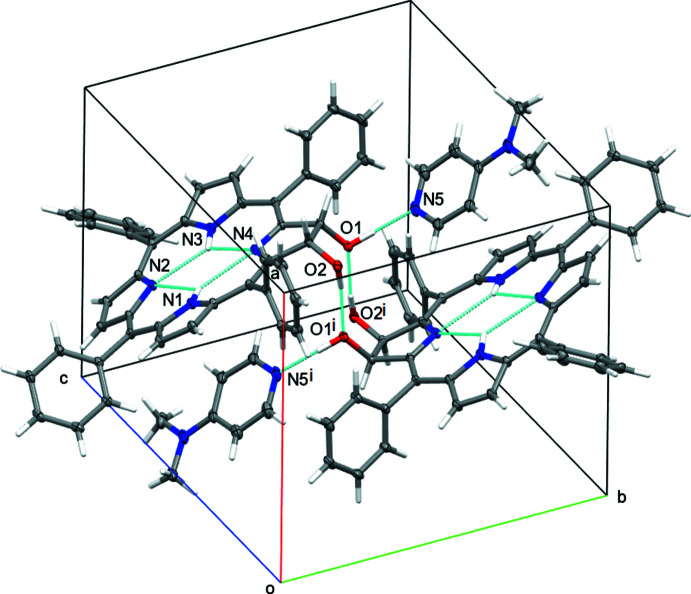
Hydrogen bonding and packing of **2^Ph^H_2_·DMAP**. 50% probability ellipsoids. Symmetry code: (i) 1 − *x*, 1 − *y*, 1 − *z*.

In **2^Ph^H_2_·DMAP** one of the hydroxyl groups acts as a donor towards the DMAP with O1—H1*O*⋯N5 = 2.6968 (14) Å. O1 in turn acts as acceptor for an O—H⋯O bond originating from O2 of a neighboring mol­ecule. A symmetry-equivalent inter­action (by inversion) connects the other two oxygen atoms of the same two mol­ecules with each other, creating an inversion-symmetric dimer (Fig. 6 ▸). A number of additional inter­actions that augment the strong hydrogen bonds, among them C—H⋯O, C—H⋯N and C–H⋯π inter­actions, are listed in the hydrogen-bonding Table 1 ▸.

**Figure 7:**
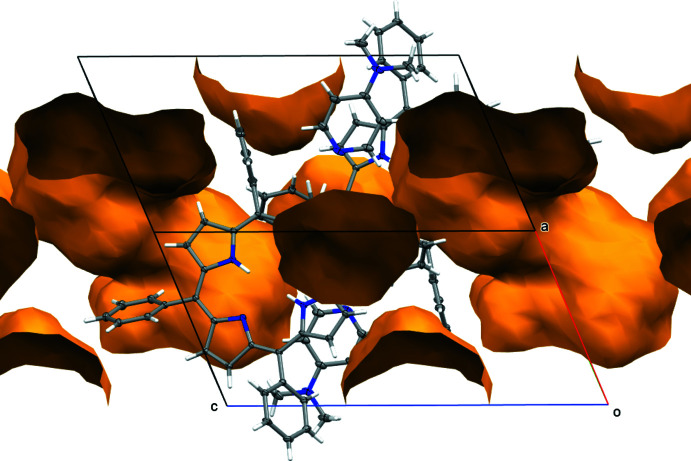
Solvent-accessible voids in **2^Ph^H_2_·DMAP**. The void volume is 647 Å^3^, or *ca* 26% of the unit-cell volume.

The structure of **2^Ph^H_2_·DMAP** also contains 647 Å^3^ (*ca* 26% of the unit-cell volume) of solvent-accessible voids occupied by highly disordered solvent mol­ecules that could not be properly modeled or refined (Fig. 7 ▸). The content of these voids, presumably chloro­form and hexane, the crystallization solvents, were instead included in the model *via* reverse-Fourier-transform methods using the SQUEEZE routine (van der Sluis & Spek, 1990 ▸; Spek, 2015 ▸) as implemented in the program *PLATON* (Spek, 2020 ▸), and added as additional not-model-based structure-factor contributions. The procedure corrected for 162 electrons within the solvent-accessible voids.

Hydrogen bonding in **2^Ph^Zn·EDA** is similar to that of **2^Ph^H_2_·DMAP**, but more complex. In contrast to the DMAP mol­ecule in **2^Ph^H_2_·DMAP**, the amino NH_2_ groups of the ethyl­ene di­amine in **2^Ph^Zn·EDA** can act as both hydrogen-bond acceptors as well as hydrogen-bond donors. One of the two amine moieties of the EDA base is axially coordinated to the zinc center of the chlorin complex, and is thus not available as a hydrogen-bond acceptor. The partially occupied methanol mol­ecule also takes part in hydrogen-bonding inter­actions, and the disorder of the not-metal-coordinated amino group further complicates the hydrogen-bonding network of **2^Ph^Zn·EDA**.

**Figure 8:**
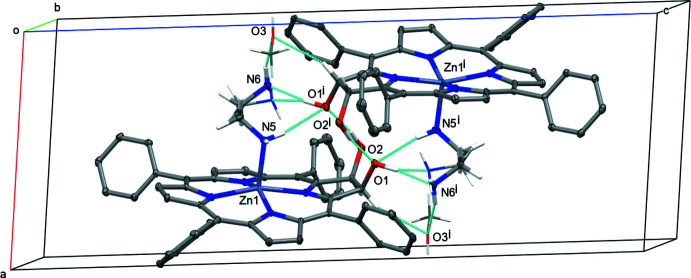
Hydrogen bonding and packing of **2^Ph^Zn·EDA**. 50% probability ellipsoids. Symmetry code: (i) 1 − *x*, 1 − *y*, 1 − *z*. 50% ellipsoids for fully occupied and major occupancy non-H atoms. Others in capped stick mode. Phenyl and pyrrole H atoms are omitted for clarity.

The two hydroxyl groups again both act as hydrogen-bond donors, and similar to in **2^Ph^H_2_·DMAP** they form an inversion-symmetric dimer (Fig. 8 ▸). O1 again acts as a hydrogen-bond donor towards the base, here the disordered amino group, of the other mol­ecule of the dimer. Different from the DMAP mol­ecule, which lacks acidic H atoms, the amines also act as hydrogen-bond donors. The metal-coordinated amine creates an N—H⋯O bond that provides an additional connection within the dimer to create a 3D hydrogen-bonding network between the two mol­ecules (Fig. 8 ▸).

Several ‘terminal’ hydrogen bonds or hydrogen-bond-like inter­actions cap off the not yet used acidic and basic atoms, which are listed in the hydrogen-bonding Table 2 ▸ (inter­actions not shown). The second amine H atom of the metal-coordin­ated NH_2_ group is engaged in an N—H⋯π inter­action towards the π-density of C29 of the phenyl ring of a neighboring mol­ecule. The major moiety of the disordered amino group hydrogen bonds with the partially occupied methanol mol­ecule. However, this inter­action is not always present, as the occupancy of the MeOH mol­ecule is only 13.6 (4)%, while that of the amino group is 88.2 (12)%. The second amino H atom is not involved in any directional inter­actions. One of the H atoms of the minor amino moiety might be engaged in another N—H⋯π inter­action towards the π-density of C43 and C43 of a phenyl ring of the second dimer mol­ecule, but the exact positions of the amino H atoms are not determined accurately given the low occupancy of the amino fragment [11.8 (12)%]. The same is true for the position of the methanol hydroxyl H atom, which appears to be engaged in a weak O—H⋯π inter­action with the porphyrinic π-system of a mol­ecule at −1 + *x*, *y*, *z*. O3, the methanol oxygen atom, acts as acceptor for a C—H⋯O inter­action originating from a phenyl C atom of a mol­ecule not part of the dimer. The H⋯O distance is unusually short for a C—H⋯O inter­action, 2.53 Å, which could be an artifact of the low occupancy of the methanol mol­ecule.

## Database survey

A search of the Cambridge Structural Database (CSD Version 5.43, Nov 2021; Groom *et al.*, 2016 ▸) for *meso*-tetra­aryl­chlorins or their metal(II) complexes revealed in excess of 75 structures, but few are directly comparable to the title compounds: Most examples contain a variety of bulky substituents or annulated rings at the pyrroline positions [the closest being an imidazolone-annulated di­hydroxy­chlorin, TAKDUI (Luciano *et al.* 2020 ▸)] or contain other (sterically encumbering) subs­tit­uents at the pyrrolic β-positions or on the *meso*-aryl groups. Most metallochlorins contain also a different metal than zinc(II). Only a few compounds are structurally closely related to **2^Ph^H_2_·DMAP** or **2^Ph^Zn·EDA**. Among them is the parent non-hy­droxy­lated chlorin zinc chelate [5,10,15,20-tetra­phenyl­chlorinato]zinc(II)·pyridine complex (HPORZN10; Spaulding *et al.*, 1977 ▸), the bis-β-*n*-butyl­ated free base and zinc(II) chlorins (QAKLUJ and QAKMAQ, respectively; Senge *et al.*, 2000 ▸), free base 5,10,15,20-tetra­phenyl-7-hy­droxy­chlorin (SAZSAP; Samankumara *et al.*, 2010 ▸), the β-nitrated analogue of **2^Ph^H_2_** (TIPBIF; Worlinsky *et al.*, 2013 ▸), dimeth­oxy derivatives **4^Ph^H_2_** (SAZROC; Samankumara *et al.*, 2010 ▸) and **4^CF3^Zn·py** (PEDKER; Sharma *et al.*, 2017 ▸), osmate ester **3^F^H_2_** (SIZFUF; Hewage *et al.*, 2019 ▸), and *trans-*7,8*-*diol-7,8-di­methyl­tetra­phenyl­chlorin (ZAZNIZ; Banerjee *et al.*, 2012 ▸).

## Synthesis and crystallization

The OsO_4_-mediated di­hydroxy­lation of porphyrin **1H_2_** is a two-step sequence: the formation of the osmate ester **3^Ar^H_2_** in the first step is followed by the reduction of the osmate ester to the target di­hydroxy­chlorin **2^Ar^H_2_** (often performed as a two-step, one-pot process) (Brückner & Dolphin, 1995*b* ▸; Samankumara *et al.*, 2010 ▸; Hyland *et al.*, 2012 ▸). Here, we prepared the inter­mediate *meso*-tetra­phenyl-2,3-*vic*-di­hydroxy­chlorin osmate ester according to the established oxidation of *meso*-tetra­phenyl­porphyrins **1^Ph^H_2_** (Brückner *et al.*, 1998 ▸). Metalation of the free base **1^Ph^H_2_** using Zn(OAc)_2_·2H_2_O under standard conditions (Buchler, 1978 ▸) (refluxing CHCl_3_/MeOH for 35-40 min) formed the corresponding Zn^II^ osmate ester **3^Ph^Zn**.

While crystallizing the osmate esters in CH_2_Cl_2_ and layering with the non-solvent hexane in the presence of DMAP (for **3^Ph^H_2_**) or by allowing a solution of the ester in CH_2_Cl_2_/MeOH to slowly evaporate in the presence of EDA (for **3^Ph^Zn**), both osmate esters adventitiously reduced and diols **2^Ph^H_2_·DMAP** and **2^Ph^Zn·EDA** crystallized, respectively. The spectroscopic data of both known chromophores are as described previously (Brückner *et al.*, 1998 ▸).

## Refinement

Crystal data, data collection and structure refinement details are summarized in Table 3 ▸. C—H bond distances were constrained to 0.95 Å for aromatic and alkene C—H groups, and to 1.00, 0.99 and 0.98 Å for aliphatic C—H, CH_2_ and CH_3_ groups, respectively. Positions of N—H and NH_2_ hydrogen atoms were refined. N—H distances within NH_2_ groups in **2^Ph^Zn·EDA** were restrained to 0.88 (2) Å and H—N—H and H–N–C angles were restrained to be similar to each other. Methyl CH_3_ and hydroxyl H atoms were allowed to rotate but not to tip to best fit the experimental electron density. The hydroxyl H atom of the partially occupied methanol mol­ecule in **2^Ph^Zn·EDA** was restrained to hydrogen bond to a porphyrin N atom of a neighboring complex. *U*_iso_(H) values were set to a multiple of *U*_eq_(C/O/N) with 1.5 for CH_3_ and OH, and 1.2 for C–H, CH_2_, N—H and NH_2_ units, respectively.

In the structure of **2^Ph^Zn·EDA**, disorder of the not-metal-coordinated amino group of the ethyl­ene di­amine mol­ecule is observed and a methanol solvate mol­ecule is partially occupied. The C—N bonds were restrained to be similar in length. A partially occupied methanol mol­ecule is located nearby the major disordered amino group and hydrogen-bonded to it. The hydroxyl H atom was restrained to hydrogen bond to a porphyrin N atom of a neighboring complex. Subject to these conditions, the occupancy ratio for the amino groups refined to 0.882 (12): 0.118 (12), and the occupancy rate for the methanol mol­ecule refined to 0.136 (4). The occupancy of the methanol mol­ecule is not correlated with the disorder of the amino group (the major 88% occupied amino group is hydrogen-bonded to the 14% occupied methanol mol­ecule).

The structure of **2^Ph^H_2_·DMAP** contains 647 Å^3^ of solvent-accessible voids occupied by highly disordered solvate mol­ecules (presumably chloro­form and hexane, the crystallization solvents). The residual electron-density peaks are not arranged in an inter­pretable pattern and no unambiguous disorder model could be developed. The structure factors were instead augmented *via* reverse-Fourier-transform methods using the SQUEEZE routine (van Sluis & Spek, 1990 ▸; Spek, 2015 ▸), as implemented in the program *PLATON* (Spek, 2020 ▸). The resultant .fab file containing the structure-factor contribution from the electron content of the void space was used in together with the original hkl file in the further refinement. The SQUEEZE procedure accounted for 162 electrons within the solvent-accessible voids.

## Supplementary Material

Crystal structure: contains datablock(s) 2PhH2, 2PhZn. DOI: 10.1107/S2056989022002729/dj2044sup1.cif

Structure factors: contains datablock(s) 2PhH2. DOI: 10.1107/S2056989022002729/dj20442PhH2sup2.hkl

Structure factors: contains datablock(s) 2PhZn. DOI: 10.1107/S2056989022002729/dj20442PhZnsup3.hkl

CCDC references: 2157745, 2157746

Additional supporting information:  crystallographic information; 3D view; checkCIF report

## Figures and Tables

**Figure 3:**
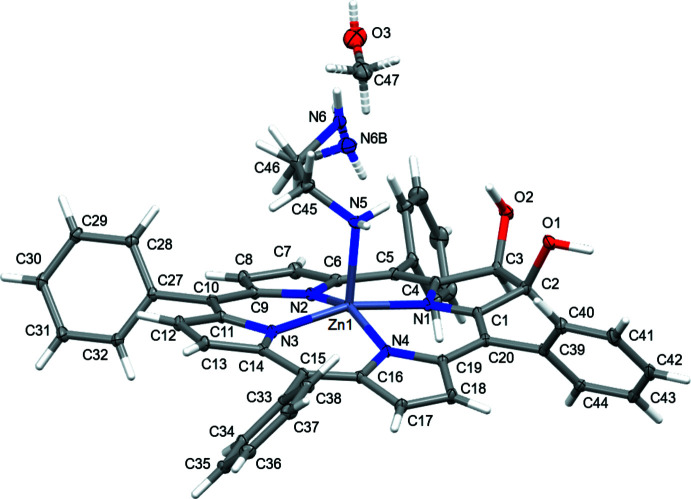
X-ray structure of the zinc(II) complex **2^Ph^Zn·EDA**, with the atom-labeling scheme for non-H atoms. 50% probability ellipsoids. Dashed bonds indicate the minor disordered amine [11.8 (12)% occupancy], and the partially occupied MeOH solvate [13.6 (4)% occupancy]. Atom labels for the backwards pointing phenyl ring (C21–C26) are omitted for clarity.

**Table 1:** Hydrogen-bond geometry (Å, °) for **2^Ph^H_2_** [Chem scheme1]

*D*—H⋯*A*	*D*—H	H⋯*A*	*D*⋯*A*	*D*—H⋯*A*
O1—H1*O*⋯N5	0.973 (17)	1.727 (17)	2.6968 (14)	174.1 (14)
O2—H2*O*⋯O1^i^	0.927 (17)	1.882 (17)	2.7798 (12)	162.5 (14)
N1—H1*N*⋯N2	0.925 (15)	2.346 (15)	2.9064 (13)	118.7 (11)
N1—H1*N*⋯N4	0.925 (15)	2.383 (15)	2.9518 (13)	119.6 (11)
N3—H3*N*⋯N2	0.915 (16)	2.292 (16)	2.8868 (13)	122.3 (12)
N3—H3*N*⋯N4	0.915 (16)	2.458 (15)	2.9766 (14)	116.1 (12)
C37—H37⋯O2^ii^	0.95	2.51	3.3840 (16)	153
C38—H38⋯C48^ii^	0.95	2.77	3.6779 (19)	161
C50—H50*B*⋯N4^ii^	0.98	2.57	3.544 (2)	171

Symmetry codes: (i) 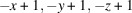 ; (ii) 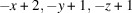 .

**Table 2:** Hydrogen-bond geometry (Å, °) for **2^Ph^Zn** [Chem scheme1]

*D*—H⋯*A*	*D*—H	H⋯*A*	*D*⋯*A*	*D*—H⋯*A*
O1—H1⋯N6^i^	0.99	1.73	2.710 (3)	168
O1—H1⋯N6*B* ^i^	0.99	1.54	2.510 (17)	165
O2—H2*A*⋯O1^i^	0.99	1.82	2.8056 (18)	171
C2—H2⋯O3^i^	1.00	2.53	3.460 (14)	155
N5—H5*A*⋯O1^i^	0.88 (2)	2.38 (2)	3.2442 (18)	166 (2)
C46—H46*A*⋯N2	0.99	2.49	3.368 (2)	148
N6—H6*A*⋯O3	0.90 (2)	2.08 (2)	2.932 (14)	159 (3)
C46*B*—H46*C*⋯N2	0.99	2.68	3.368 (2)	126
O3—H3*O*⋯N4^ii^	0.84	2.20	2.992 (14)	157

Symmetry codes: (i) 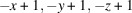 ; (ii) 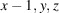 .

**Table 3:** Experimental details

	**2^Ph^H_2_**	**2^Ph^Zn**
Crystal data		
Chemical formula	C_44_H_32_N_4_O_2_·C_7_H_10_N_2_·[+solvent]	[Zn(C_44_H_30_N_4_O_2_)]·C_2_H_8_N_2_·0.136CH_4_O
*M*_r_	770.90	776.57
Crystal system, space group	Triclinic, *P* 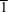	Monoclinic, *P*2_1_/*c*
Temperature (K)	150	150
*a*, *b*, *c* (Å)	10.0193 (4), 15.2554 (8), 17.7983 (10)	10.1249 (3), 13.5400 (4), 27.0447 (8)
α, β, γ (°)	69.918 (2), 74.926 (2), 84.140 (2)	90, 95.1464 (11), 90
*V* (Å^3^)	2466.9 (2)	3692.64 (19)
*Z*	2	4
Radiation type	Mo *K*α	Cu *K*α
μ (mm^−1^)	0.06	1.32
Crystal size (mm)	0.33 × 0.21 × 0.19	0.27 × 0.25 × 0.18

Data collection		
Diffractometer	Bruker AXS D8 Quest diffractometer with PhotonII charge-integrating pixel array detector (CPAD)	Bruker AXS D8 Quest diffractometer with PhotonIII-C14 charge-integrating and photon counting pixel array detector
Absorption correction	Multi-scan (*SADABS*; Krause *et al.*, 2015 ▸)	Multi-scan (*SADABS*; Krause *et al.*, 2015 ▸)
*T*_min_, *T*_max_	0.665, 0.746	0.606, 0.754
No. of measured, independent and observed [*I* > 2σ(*I*)] reflections	48645, 14738, 9891	21319, 7551, 7037
*R*_int_	0.060	0.024
(sin θ/λ)_max_ (Å^−1^)	0.714	0.638

Refinement		
*R*[*F* ^2^ > 2σ(*F* ^2^)], *wR*(*F* ^2^), *S*	0.048, 0.133, 1.04	0.031, 0.088, 1.04
No. of reflections	14738	7551
No. of parameters	549	549
No. of restraints	0	17
H-atom treatment	H atoms treated by a mixture of independent and constrained refinement	H atoms treated by a mixture of independent and constrained refinement
Δρ_max_, Δρ_min_ (e Å^−3^)	0.45, −0.21	0.31, −0.44

Computer programs: *APEX4* (Bruker, 2021 ▸), *APEX3* and *SAINT* (Bruker, 2019 ▸), *SHELXT* (Sheldrick, 2015*a* ▸), *SHELXS97* (Sheldrick, 2008 ▸), *SHELXL2018/3* (Sheldrick, 2015*b* ▸), *ShelXle* (Hübschle *et al.*, 2011 ▸), *Mercury* (Macrae *et al.*, 2020 ▸), and *publCIF* (Westrip, 2010 ▸).

## supplementary crystallographic information

### cis-7,8-Dihydroxy-5,10,15,20-tetraphenylchlorin dimethylaminopyridine monosolvate (2PhH2) Crystal data

**Table:**

C_44_H_32_N_4_O_2_·C_7_H_10_N_2_·[+solvent]	*Z* = 2
*M**_r_* = 770.90	*F*(000) = 812
Triclinic, *P*1	*D*_x_ = 1.038 Mg m^−^^3^
*a* = 10.0193 (4) Å	Mo *K*α radiation, λ = 0.71073 Å
*b* = 15.2554 (8) Å	Cell parameters from 9960 reflections
*c* = 17.7983 (10) Å	θ = 2.4–31.9°
α = 69.918 (2)°	µ = 0.06 mm^−^^1^
β = 74.926 (2)°	*T* = 150 K
γ = 84.140 (2)°	Fragment, black
*V* = 2466.9 (2) Å^3^	0.33 × 0.21 × 0.19 mm

### cis-7,8-Dihydroxy-5,10,15,20-tetraphenylchlorin dimethylaminopyridine monosolvate (2PhH2) Data collection

**Table:**

Bruker AXS D8 Quest diffractometer with PhotonII charge-integrating pixel array detector (CPAD)	14738 independent reflections
Radiation source: fine focus sealed tube X-ray source	9891 reflections with *I* > 2σ(*I*)
Triumph curved graphite crystal monochromator	*R*_int_ = 0.060
Detector resolution: 7.4074 pixels mm^-1^	θ_max_ = 30.5°, θ_min_ = 2.2°
ω and phi scans	*h* = −14→14
Absorption correction: multi-scan (SADABS; Krause *et al.*, 2015)	*k* = −21→21
*T*_min_ = 0.665, *T*_max_ = 0.746	*l* = −25→25
48645 measured reflections	

### cis-7,8-Dihydroxy-5,10,15,20-tetraphenylchlorin dimethylaminopyridine monosolvate (2PhH2) Refinement

**Table:**

Refinement on *F*^2^	Primary atom site location: dual
Least-squares matrix: full	Secondary atom site location: difference Fourier map
*R*[*F*^2^ > 2σ(*F*^2^)] = 0.048	Hydrogen site location: mixed
*wR*(*F*^2^) = 0.133	H atoms treated by a mixture of independent and constrained refinement
*S* = 1.04	*w* = 1/[σ^2^(*F*_o_^2^) + (0.0604*P*)^2^ + 0.2687*P*] where *P* = (*F*_o_^2^ + 2*F*_c_^2^)/3
14738 reflections	(Δ/σ)_max_ = 0.001
549 parameters	Δρ_max_ = 0.45 e Å^−^^3^
0 restraints	Δρ_min_ = −0.21 e Å^−^^3^

### cis-7,8-Dihydroxy-5,10,15,20-tetraphenylchlorin dimethylaminopyridine monosolvate (2PhH2) Special details

**Table:**

Geometry. All esds (except the esd in the dihedral angle between two l.s. planes) are estimated using the full covariance matrix. The cell esds are taken into account individually in the estimation of esds in distances, angles and torsion angles; correlations between esds in cell parameters are only used when they are defined by crystal symmetry. An approximate (isotropic) treatment of cell esds is used for estimating esds involving l.s. planes.
Refinement. The structure contains 647 Ang3 of solvent accessible voids occupied by highly disordered solvate molecules (presumably chloroform and hexane, the crystallization solvents). The residual electron density peaks are not arranged in an interpretable pattern and no unambiguous disorder model could be developed. The structure factors were instead augmented via reverse Fourier transform methods using the SQUEEZE routine (P. van der Sluis & A.L. Spek (1990). Acta Cryst. A46, 194-201) as implemented in the program Platon. The resultant FAB file containing the structure factor contribution from the electron content of the void space was used in together with the original hkl file in the further refinement. (The FAB file with details of the Squeeze results is appended to this cif file). The Squeeze procedure corrected for 162 electrons within the solvent accessible voids.

### cis-7,8-Dihydroxy-5,10,15,20-tetraphenylchlorin dimethylaminopyridine monosolvate (2PhH2) Fractional atomic coordinates and isotropic or equivalent isotropic displacement parameters (Å^2^)

**Table:**

	*x*	*y*	*z*	*U*_iso_*/*U*_eq_	
O1	0.62197 (8)	0.52783 (6)	0.53614 (5)	0.03032 (18)	
H1O	0.6812 (16)	0.5788 (11)	0.4973 (10)	0.045*	
O2	0.65897 (9)	0.42954 (6)	0.42876 (5)	0.03175 (18)	
H2O	0.5704 (17)	0.4513 (11)	0.4458 (10)	0.048*	
N1	0.43218 (10)	0.14254 (6)	0.68820 (6)	0.02595 (19)	
H1N	0.4495 (15)	0.1795 (10)	0.7162 (9)	0.039*	
N2	0.37812 (10)	0.12770 (6)	0.86064 (6)	0.02649 (19)	
N3	0.55460 (10)	0.28307 (7)	0.82259 (6)	0.0283 (2)	
H3N	0.5129 (16)	0.2577 (11)	0.7949 (10)	0.042*	
N4	0.59891 (9)	0.30953 (6)	0.64424 (6)	0.02597 (19)	
N5	0.79133 (13)	0.66232 (8)	0.42197 (8)	0.0513 (3)	
N6	1.09618 (14)	0.82611 (9)	0.22875 (8)	0.0542 (3)	
C1	0.48576 (12)	0.15869 (8)	0.60568 (7)	0.0272 (2)	
C2	0.43926 (14)	0.08451 (8)	0.58748 (8)	0.0344 (3)	
H2	0.461662	0.076127	0.535118	0.041*	
C3	0.35751 (13)	0.02790 (8)	0.65773 (7)	0.0329 (3)	
H3	0.312720	−0.026520	0.662782	0.039*	
C4	0.35068 (11)	0.06429 (7)	0.72235 (7)	0.0261 (2)	
C5	0.27900 (11)	0.02833 (7)	0.80485 (7)	0.0258 (2)	
C6	0.28682 (11)	0.06218 (8)	0.86799 (7)	0.0258 (2)	
C7	0.20051 (12)	0.02932 (8)	0.95097 (7)	0.0292 (2)	
H7	0.128291	−0.014687	0.970852	0.035*	
C8	0.24279 (12)	0.07350 (8)	0.99458 (7)	0.0300 (2)	
H8	0.206040	0.066657	1.051130	0.036*	
C9	0.35477 (11)	0.13318 (8)	0.93846 (7)	0.0270 (2)	
C10	0.42982 (12)	0.18860 (8)	0.96202 (7)	0.0282 (2)	
C11	0.52658 (12)	0.25542 (8)	0.90752 (7)	0.0293 (2)	
C12	0.60953 (13)	0.31160 (9)	0.92638 (8)	0.0349 (3)	
H12	0.614596	0.307363	0.980081	0.042*	
C13	0.68056 (13)	0.37261 (9)	0.85413 (8)	0.0344 (3)	
H13	0.742769	0.418500	0.848983	0.041*	
C14	0.64544 (12)	0.35568 (8)	0.78779 (7)	0.0290 (2)	
C15	0.69121 (11)	0.40654 (7)	0.70330 (7)	0.0266 (2)	
C16	0.66552 (11)	0.38612 (7)	0.63837 (7)	0.0256 (2)	
C17	0.71366 (11)	0.44961 (8)	0.54998 (7)	0.0269 (2)	
H17	0.810994	0.469724	0.538272	0.032*	
C18	0.70357 (11)	0.38542 (8)	0.50137 (7)	0.0271 (2)	
H18	0.798257	0.359268	0.485428	0.033*	
C19	0.61540 (11)	0.30561 (7)	0.56736 (7)	0.0258 (2)	
C20	0.56811 (11)	0.23380 (8)	0.54904 (7)	0.0267 (2)	
C21	0.18885 (12)	−0.05389 (8)	0.82851 (7)	0.0277 (2)	
C22	0.21160 (13)	−0.13703 (8)	0.88883 (8)	0.0340 (3)	
H22	0.286768	−0.142088	0.913483	0.041*	
C23	0.12514 (15)	−0.21243 (9)	0.91308 (9)	0.0418 (3)	
H23	0.141490	−0.268761	0.954185	0.050*	
C24	0.01525 (14)	−0.20589 (10)	0.87763 (9)	0.0428 (3)	
H24	−0.044311	−0.257364	0.894713	0.051*	
C25	−0.00731 (13)	−0.12454 (10)	0.81758 (9)	0.0392 (3)	
H25	−0.082307	−0.120161	0.792940	0.047*	
C26	0.07881 (12)	−0.04862 (9)	0.79264 (8)	0.0321 (2)	
H26	0.062527	0.007135	0.750918	0.039*	
C27	0.40379 (12)	0.17673 (9)	1.05123 (7)	0.0306 (2)	
C28	0.42357 (13)	0.09033 (9)	1.10829 (8)	0.0350 (3)	
H28	0.453285	0.038015	1.090474	0.042*	
C29	0.39991 (14)	0.08048 (11)	1.19136 (8)	0.0438 (3)	
H29	0.413317	0.021333	1.230008	0.053*	
C30	0.35704 (17)	0.15613 (13)	1.21802 (9)	0.0518 (4)	
H30	0.341547	0.149010	1.274766	0.062*	
C31	0.33677 (18)	0.24209 (12)	1.16204 (9)	0.0532 (4)	
H31	0.307152	0.294134	1.180274	0.064*	
C32	0.35962 (15)	0.25240 (10)	1.07935 (8)	0.0409 (3)	
H32	0.345099	0.311659	1.041196	0.049*	
C33	0.77693 (12)	0.48962 (8)	0.68495 (7)	0.0277 (2)	
C34	0.71456 (13)	0.57598 (9)	0.68111 (8)	0.0357 (3)	
H34	0.617350	0.583199	0.687579	0.043*	
C35	0.79380 (15)	0.65217 (10)	0.66780 (9)	0.0435 (3)	
H35	0.750570	0.711286	0.664713	0.052*	
C36	0.93499 (15)	0.64211 (10)	0.65908 (9)	0.0443 (3)	
H36	0.988404	0.693901	0.651294	0.053*	
C37	0.99855 (14)	0.55681 (11)	0.66166 (9)	0.0444 (3)	
H37	1.095810	0.549926	0.655054	0.053*	
C38	0.91967 (13)	0.48109 (9)	0.67397 (9)	0.0379 (3)	
H38	0.963808	0.422699	0.674888	0.045*	
C39	0.61114 (12)	0.23285 (8)	0.46200 (7)	0.0285 (2)	
C40	0.51769 (14)	0.25180 (9)	0.41304 (8)	0.0353 (3)	
H40	0.424591	0.267589	0.434044	0.042*	
C41	0.55892 (15)	0.24792 (10)	0.33351 (8)	0.0408 (3)	
H41	0.494024	0.261266	0.300357	0.049*	
C42	0.69418 (15)	0.22468 (9)	0.30227 (8)	0.0405 (3)	
H42	0.722094	0.221702	0.247937	0.049*	
C43	0.78742 (14)	0.20604 (10)	0.35014 (9)	0.0424 (3)	
H43	0.880424	0.190326	0.328850	0.051*	
C44	0.74671 (13)	0.20998 (9)	0.42957 (8)	0.0369 (3)	
H44	0.812284	0.196878	0.462273	0.044*	
C45	0.89678 (18)	0.69732 (11)	0.43549 (10)	0.0546 (4)	
H45	0.901928	0.682842	0.491021	0.065*	
C46	0.99821 (17)	0.75277 (10)	0.37465 (10)	0.0504 (4)	
H46	1.069780	0.775754	0.388603	0.060*	
C47	0.99487 (15)	0.77500 (9)	0.29192 (9)	0.0435 (3)	
C48	0.88343 (15)	0.73985 (10)	0.27753 (10)	0.0484 (4)	
H48	0.873990	0.753889	0.222851	0.058*	
C49	0.78732 (15)	0.68461 (10)	0.34342 (11)	0.0504 (4)	
H49	0.713560	0.661011	0.331852	0.060*	
C50	1.20258 (17)	0.86988 (12)	0.24494 (12)	0.0637 (5)	
H50A	1.267695	0.901455	0.192839	0.096*	
H50B	1.252073	0.822161	0.281093	0.096*	
H50C	1.159873	0.915591	0.271836	0.096*	
C51	1.09541 (19)	0.84148 (13)	0.14456 (10)	0.0656 (5)	
H51A	1.184094	0.867545	0.108436	0.098*	
H51B	1.020473	0.885300	0.129652	0.098*	
H51C	1.081150	0.782071	0.138165	0.098*	

### cis-7,8-Dihydroxy-5,10,15,20-tetraphenylchlorin dimethylaminopyridine monosolvate (2PhH2) Atomic displacement parameters (Å^2^)

**Table:**

	*U*^11^	*U*^22^	*U*^33^	*U*^12^	*U*^13^	*U*^23^
O1	0.0308 (4)	0.0229 (4)	0.0316 (4)	−0.0034 (3)	−0.0017 (3)	−0.0053 (3)
O2	0.0346 (4)	0.0310 (4)	0.0244 (4)	−0.0029 (4)	−0.0022 (3)	−0.0055 (3)
N1	0.0303 (5)	0.0227 (4)	0.0226 (4)	−0.0058 (4)	−0.0006 (4)	−0.0074 (4)
N2	0.0297 (5)	0.0251 (4)	0.0228 (5)	−0.0050 (4)	−0.0019 (4)	−0.0075 (4)
N3	0.0328 (5)	0.0262 (5)	0.0241 (5)	−0.0088 (4)	−0.0024 (4)	−0.0070 (4)
N4	0.0278 (4)	0.0240 (4)	0.0237 (5)	−0.0044 (4)	−0.0022 (4)	−0.0068 (4)
N5	0.0458 (7)	0.0300 (6)	0.0536 (8)	−0.0030 (5)	0.0108 (6)	0.0007 (5)
N6	0.0464 (7)	0.0388 (6)	0.0517 (8)	−0.0073 (6)	0.0050 (6)	0.0067 (6)
C1	0.0315 (5)	0.0248 (5)	0.0230 (5)	−0.0046 (4)	−0.0009 (4)	−0.0078 (4)
C2	0.0451 (7)	0.0297 (6)	0.0270 (6)	−0.0103 (5)	0.0010 (5)	−0.0120 (5)
C3	0.0430 (6)	0.0273 (5)	0.0275 (6)	−0.0102 (5)	−0.0015 (5)	−0.0103 (5)
C4	0.0301 (5)	0.0214 (5)	0.0244 (5)	−0.0051 (4)	−0.0025 (4)	−0.0062 (4)
C5	0.0265 (5)	0.0230 (5)	0.0256 (5)	−0.0041 (4)	−0.0028 (4)	−0.0066 (4)
C6	0.0267 (5)	0.0248 (5)	0.0231 (5)	−0.0036 (4)	−0.0025 (4)	−0.0061 (4)
C7	0.0281 (5)	0.0315 (6)	0.0243 (5)	−0.0077 (5)	−0.0002 (4)	−0.0072 (5)
C8	0.0309 (5)	0.0335 (6)	0.0222 (5)	−0.0056 (5)	0.0002 (4)	−0.0082 (5)
C9	0.0287 (5)	0.0258 (5)	0.0240 (5)	−0.0027 (4)	−0.0026 (4)	−0.0071 (4)
C10	0.0323 (5)	0.0263 (5)	0.0240 (5)	−0.0040 (4)	−0.0032 (4)	−0.0073 (4)
C11	0.0339 (6)	0.0281 (5)	0.0250 (5)	−0.0048 (5)	−0.0044 (5)	−0.0083 (4)
C12	0.0417 (7)	0.0355 (6)	0.0280 (6)	−0.0111 (5)	−0.0069 (5)	−0.0091 (5)
C13	0.0386 (6)	0.0351 (6)	0.0308 (6)	−0.0118 (5)	−0.0062 (5)	−0.0107 (5)
C14	0.0324 (5)	0.0251 (5)	0.0285 (6)	−0.0063 (4)	−0.0035 (5)	−0.0087 (4)
C15	0.0277 (5)	0.0227 (5)	0.0270 (5)	−0.0051 (4)	−0.0025 (4)	−0.0069 (4)
C16	0.0252 (5)	0.0229 (5)	0.0250 (5)	−0.0037 (4)	−0.0020 (4)	−0.0053 (4)
C17	0.0256 (5)	0.0242 (5)	0.0256 (5)	−0.0048 (4)	−0.0006 (4)	−0.0044 (4)
C18	0.0264 (5)	0.0264 (5)	0.0238 (5)	−0.0039 (4)	0.0002 (4)	−0.0059 (4)
C19	0.0256 (5)	0.0238 (5)	0.0236 (5)	−0.0023 (4)	−0.0004 (4)	−0.0059 (4)
C20	0.0290 (5)	0.0251 (5)	0.0227 (5)	−0.0032 (4)	−0.0009 (4)	−0.0071 (4)
C21	0.0289 (5)	0.0266 (5)	0.0256 (5)	−0.0062 (4)	0.0013 (4)	−0.0102 (4)
C22	0.0375 (6)	0.0303 (6)	0.0303 (6)	−0.0087 (5)	−0.0032 (5)	−0.0063 (5)
C23	0.0463 (7)	0.0291 (6)	0.0406 (7)	−0.0117 (5)	0.0022 (6)	−0.0059 (5)
C24	0.0380 (7)	0.0378 (7)	0.0496 (8)	−0.0173 (6)	0.0082 (6)	−0.0198 (6)
C25	0.0302 (6)	0.0460 (7)	0.0463 (8)	−0.0084 (5)	−0.0011 (6)	−0.0252 (6)
C26	0.0308 (6)	0.0334 (6)	0.0328 (6)	−0.0036 (5)	−0.0025 (5)	−0.0146 (5)
C27	0.0324 (6)	0.0347 (6)	0.0242 (5)	−0.0088 (5)	−0.0044 (5)	−0.0084 (5)
C28	0.0312 (6)	0.0395 (7)	0.0312 (6)	−0.0064 (5)	−0.0076 (5)	−0.0061 (5)
C29	0.0402 (7)	0.0565 (9)	0.0297 (7)	−0.0134 (6)	−0.0135 (6)	−0.0006 (6)
C30	0.0581 (9)	0.0730 (11)	0.0286 (7)	−0.0243 (8)	−0.0086 (7)	−0.0167 (7)
C31	0.0709 (10)	0.0573 (9)	0.0370 (8)	−0.0205 (8)	−0.0013 (7)	−0.0256 (7)
C32	0.0537 (8)	0.0374 (7)	0.0320 (7)	−0.0110 (6)	−0.0038 (6)	−0.0139 (5)
C33	0.0295 (5)	0.0276 (5)	0.0239 (5)	−0.0075 (4)	−0.0009 (4)	−0.0079 (4)
C34	0.0327 (6)	0.0314 (6)	0.0412 (7)	−0.0070 (5)	0.0012 (5)	−0.0153 (5)
C35	0.0499 (8)	0.0330 (6)	0.0470 (8)	−0.0116 (6)	0.0020 (6)	−0.0193 (6)
C36	0.0509 (8)	0.0459 (8)	0.0363 (7)	−0.0257 (7)	−0.0007 (6)	−0.0140 (6)
C37	0.0332 (6)	0.0545 (8)	0.0392 (7)	−0.0189 (6)	−0.0025 (6)	−0.0072 (6)
C38	0.0301 (6)	0.0364 (6)	0.0413 (7)	−0.0054 (5)	−0.0036 (5)	−0.0076 (6)
C39	0.0349 (6)	0.0239 (5)	0.0237 (5)	−0.0080 (4)	0.0015 (5)	−0.0081 (4)
C40	0.0376 (6)	0.0368 (6)	0.0296 (6)	−0.0028 (5)	−0.0023 (5)	−0.0121 (5)
C41	0.0533 (8)	0.0402 (7)	0.0308 (7)	−0.0056 (6)	−0.0090 (6)	−0.0134 (6)
C42	0.0548 (8)	0.0362 (7)	0.0292 (6)	−0.0141 (6)	0.0052 (6)	−0.0165 (5)
C43	0.0393 (7)	0.0483 (8)	0.0418 (7)	−0.0106 (6)	0.0071 (6)	−0.0272 (7)
C44	0.0352 (6)	0.0403 (7)	0.0374 (7)	−0.0039 (5)	−0.0009 (5)	−0.0204 (6)
C45	0.0650 (10)	0.0410 (8)	0.0440 (8)	−0.0082 (7)	0.0077 (8)	−0.0101 (7)
C46	0.0550 (9)	0.0366 (7)	0.0508 (9)	−0.0091 (6)	0.0013 (7)	−0.0117 (6)
C47	0.0415 (7)	0.0234 (6)	0.0466 (8)	−0.0005 (5)	0.0051 (6)	0.0006 (5)
C48	0.0431 (8)	0.0367 (7)	0.0486 (8)	0.0037 (6)	−0.0048 (6)	0.0013 (6)
C49	0.0368 (7)	0.0340 (7)	0.0629 (10)	0.0013 (6)	−0.0042 (7)	−0.0005 (7)
C50	0.0418 (8)	0.0402 (8)	0.0829 (13)	−0.0060 (7)	−0.0022 (8)	0.0046 (8)
C51	0.0553 (10)	0.0607 (10)	0.0467 (9)	0.0059 (8)	0.0061 (8)	0.0095 (8)

### cis-7,8-Dihydroxy-5,10,15,20-tetraphenylchlorin dimethylaminopyridine monosolvate (2PhH2) Geometric parameters (Å, º)

**Table:**

O1—C17	1.4214 (14)	C23—C24	1.384 (2)
O1—H1O	0.973 (17)	C23—H23	0.9500
O2—C18	1.4016 (14)	C24—C25	1.375 (2)
O2—H2O	0.927 (17)	C24—H24	0.9500
N1—C1	1.3692 (14)	C25—C26	1.3909 (17)
N1—C4	1.3797 (14)	C25—H25	0.9500
N1—H1N	0.925 (15)	C26—H26	0.9500
N2—C9	1.3737 (14)	C27—C28	1.3929 (18)
N2—C6	1.3740 (14)	C27—C32	1.3986 (17)
N3—C14	1.3714 (14)	C28—C29	1.3916 (18)
N3—C11	1.3811 (15)	C28—H28	0.9500
N3—H3N	0.915 (16)	C29—C30	1.382 (2)
N4—C19	1.3565 (14)	C29—H29	0.9500
N4—C16	1.3639 (14)	C30—C31	1.381 (2)
N5—C49	1.331 (2)	C30—H30	0.9500
N5—C45	1.340 (2)	C31—C32	1.3843 (19)
N6—C47	1.3669 (18)	C31—H31	0.9500
N6—C51	1.437 (2)	C32—H32	0.9500
N6—C50	1.450 (2)	C33—C34	1.3867 (17)
C1—C20	1.4062 (15)	C33—C38	1.3910 (17)
C1—C2	1.4284 (16)	C34—C35	1.3924 (17)
C2—C3	1.3602 (17)	C34—H34	0.9500
C2—H2	0.9500	C35—C36	1.381 (2)
C3—C4	1.4232 (16)	C35—H35	0.9500
C3—H3	0.9500	C36—C37	1.381 (2)
C4—C5	1.3970 (15)	C36—H36	0.9500
C5—C6	1.4101 (16)	C37—C38	1.3891 (19)
C5—C21	1.4958 (15)	C37—H37	0.9500
C6—C7	1.4478 (16)	C38—H38	0.9500
C7—C8	1.3508 (16)	C39—C40	1.3840 (18)
C7—H7	0.9500	C39—C44	1.3905 (17)
C8—C9	1.4445 (16)	C40—C41	1.3879 (17)
C8—H8	0.9500	C40—H40	0.9500
C9—C10	1.4119 (16)	C41—C42	1.384 (2)
C10—C11	1.3977 (16)	C41—H41	0.9500
C10—C27	1.4895 (16)	C42—C43	1.368 (2)
C11—C12	1.4233 (16)	C42—H42	0.9500
C12—C13	1.3651 (18)	C43—C44	1.3864 (18)
C12—H12	0.9500	C43—H43	0.9500
C13—C14	1.4243 (17)	C44—H44	0.9500
C13—H13	0.9500	C45—C46	1.377 (2)
C14—C15	1.4094 (16)	C45—H45	0.9500
C15—C16	1.3846 (16)	C46—C47	1.402 (2)
C15—C33	1.4977 (15)	C46—H46	0.9500
C16—C17	1.5166 (16)	C47—C48	1.402 (2)
C17—C18	1.5380 (16)	C48—C49	1.383 (2)
C17—H17	1.0000	C48—H48	0.9500
C18—C19	1.5298 (15)	C49—H49	0.9500
C18—H18	1.0000	C50—H50A	0.9800
C19—C20	1.4008 (15)	C50—H50B	0.9800
C20—C39	1.5008 (15)	C50—H50C	0.9800
C21—C26	1.3937 (17)	C51—H51A	0.9800
C21—C22	1.3951 (17)	C51—H51B	0.9800
C22—C23	1.3884 (17)	C51—H51C	0.9800
C22—H22	0.9500		
			
C17—O1—H1O	103.8 (9)	C25—C24—H24	120.2
C18—O2—H2O	105.0 (10)	C23—C24—H24	120.2
C1—N1—C4	110.47 (9)	C24—C25—C26	120.52 (13)
C1—N1—H1N	123.8 (9)	C24—C25—H25	119.7
C4—N1—H1N	125.7 (9)	C26—C25—H25	119.7
C9—N2—C6	104.78 (9)	C25—C26—C21	120.38 (12)
C14—N3—C11	110.47 (10)	C25—C26—H26	119.8
C14—N3—H3N	126.2 (10)	C21—C26—H26	119.8
C11—N3—H3N	123.2 (10)	C28—C27—C32	118.61 (12)
C19—N4—C16	108.59 (9)	C28—C27—C10	120.80 (11)
C49—N5—C45	115.86 (13)	C32—C27—C10	120.58 (11)
C47—N6—C51	120.56 (15)	C29—C28—C27	120.15 (13)
C47—N6—C50	120.85 (15)	C29—C28—H28	119.9
C51—N6—C50	118.52 (14)	C27—C28—H28	119.9
N1—C1—C20	127.44 (10)	C30—C29—C28	120.47 (14)
N1—C1—C2	106.31 (10)	C30—C29—H29	119.8
C20—C1—C2	126.24 (10)	C28—C29—H29	119.8
C3—C2—C1	108.53 (10)	C31—C30—C29	119.90 (13)
C3—C2—H2	125.7	C31—C30—H30	120.1
C1—C2—H2	125.7	C29—C30—H30	120.1
C2—C3—C4	108.18 (10)	C30—C31—C32	119.99 (14)
C2—C3—H3	125.9	C30—C31—H31	120.0
C4—C3—H3	125.9	C32—C31—H31	120.0
N1—C4—C5	125.72 (10)	C31—C32—C27	120.88 (14)
N1—C4—C3	106.47 (10)	C31—C32—H32	119.6
C5—C4—C3	127.81 (10)	C27—C32—H32	119.6
C4—C5—C6	125.26 (10)	C34—C33—C38	118.83 (11)
C4—C5—C21	117.43 (10)	C34—C33—C15	120.39 (10)
C6—C5—C21	117.30 (10)	C38—C33—C15	120.75 (11)
N2—C6—C5	125.47 (10)	C33—C34—C35	120.29 (12)
N2—C6—C7	110.91 (9)	C33—C34—H34	119.9
C5—C6—C7	123.59 (10)	C35—C34—H34	119.9
C8—C7—C6	106.56 (10)	C36—C35—C34	120.24 (13)
C8—C7—H7	126.7	C36—C35—H35	119.9
C6—C7—H7	126.7	C34—C35—H35	119.9
C7—C8—C9	106.62 (10)	C37—C36—C35	120.02 (12)
C7—C8—H8	126.7	C37—C36—H36	120.0
C9—C8—H8	126.7	C35—C36—H36	120.0
N2—C9—C10	125.32 (10)	C36—C37—C38	119.72 (13)
N2—C9—C8	111.05 (10)	C36—C37—H37	120.1
C10—C9—C8	123.63 (10)	C38—C37—H37	120.1
C11—C10—C9	124.84 (11)	C37—C38—C33	120.87 (13)
C11—C10—C27	116.86 (10)	C37—C38—H38	119.6
C9—C10—C27	118.30 (10)	C33—C38—H38	119.6
N3—C11—C10	125.39 (10)	C40—C39—C44	118.43 (11)
N3—C11—C12	106.27 (10)	C40—C39—C20	121.53 (10)
C10—C11—C12	128.27 (11)	C44—C39—C20	120.02 (11)
C13—C12—C11	108.35 (11)	C39—C40—C41	120.53 (12)
C13—C12—H12	125.8	C39—C40—H40	119.7
C11—C12—H12	125.8	C41—C40—H40	119.7
C12—C13—C14	108.32 (11)	C42—C41—C40	120.31 (13)
C12—C13—H13	125.8	C42—C41—H41	119.8
C14—C13—H13	125.8	C40—C41—H41	119.8
N3—C14—C15	127.08 (10)	C43—C42—C41	119.60 (12)
N3—C14—C13	106.53 (10)	C43—C42—H42	120.2
C15—C14—C13	126.34 (10)	C41—C42—H42	120.2
C16—C15—C14	126.44 (10)	C42—C43—C44	120.28 (12)
C16—C15—C33	118.94 (10)	C42—C43—H43	119.9
C14—C15—C33	114.62 (10)	C44—C43—H43	119.9
N4—C16—C15	126.10 (10)	C43—C44—C39	120.86 (13)
N4—C16—C17	112.44 (9)	C43—C44—H44	119.6
C15—C16—C17	121.45 (10)	C39—C44—H44	119.6
O1—C17—C16	108.57 (9)	N5—C45—C46	124.66 (17)
O1—C17—C18	112.64 (9)	N5—C45—H45	117.7
C16—C17—C18	101.86 (9)	C46—C45—H45	117.7
O1—C17—H17	111.1	C45—C46—C47	119.36 (16)
C16—C17—H17	111.1	C45—C46—H46	120.3
C18—C17—H17	111.1	C47—C46—H46	120.3
O2—C18—C19	117.07 (9)	N6—C47—C46	121.97 (15)
O2—C18—C17	115.00 (9)	N6—C47—C48	121.83 (15)
C19—C18—C17	102.30 (9)	C46—C47—C48	116.18 (13)
O2—C18—H18	107.3	C49—C48—C47	119.59 (16)
C19—C18—H18	107.3	C49—C48—H48	120.2
C17—C18—H18	107.3	C47—C48—H48	120.2
N4—C19—C20	125.15 (10)	N5—C49—C48	124.33 (16)
N4—C19—C18	112.16 (9)	N5—C49—H49	117.8
C20—C19—C18	122.51 (10)	C48—C49—H49	117.8
C19—C20—C1	126.08 (10)	N6—C50—H50A	109.5
C19—C20—C39	118.64 (10)	N6—C50—H50B	109.5
C1—C20—C39	115.24 (10)	H50A—C50—H50B	109.5
C26—C21—C22	118.64 (11)	N6—C50—H50C	109.5
C26—C21—C5	121.07 (11)	H50A—C50—H50C	109.5
C22—C21—C5	120.27 (11)	H50B—C50—H50C	109.5
C23—C22—C21	120.46 (12)	N6—C51—H51A	109.5
C23—C22—H22	119.8	N6—C51—H51B	109.5
C21—C22—H22	119.8	H51A—C51—H51B	109.5
C24—C23—C22	120.31 (13)	N6—C51—H51C	109.5
C24—C23—H23	119.8	H51A—C51—H51C	109.5
C22—C23—H23	119.8	H51B—C51—H51C	109.5
C25—C24—C23	119.69 (12)		
			
C4—N1—C1—C20	176.83 (11)	C18—C19—C20—C1	−179.31 (11)
C4—N1—C1—C2	−2.11 (13)	N4—C19—C20—C39	−171.75 (10)
N1—C1—C2—C3	1.49 (14)	C18—C19—C20—C39	2.90 (16)
C20—C1—C2—C3	−177.46 (12)	N1—C1—C20—C19	2.4 (2)
C1—C2—C3—C4	−0.35 (15)	C2—C1—C20—C19	−178.88 (12)
C1—N1—C4—C5	−179.30 (11)	N1—C1—C20—C39	−179.76 (11)
C1—N1—C4—C3	1.91 (13)	C2—C1—C20—C39	−1.02 (18)
C2—C3—C4—N1	−0.92 (14)	C4—C5—C21—C26	−60.11 (15)
C2—C3—C4—C5	−179.68 (12)	C6—C5—C21—C26	121.08 (12)
N1—C4—C5—C6	−5.13 (19)	C4—C5—C21—C22	121.51 (12)
C3—C4—C5—C6	173.41 (12)	C6—C5—C21—C22	−57.30 (15)
N1—C4—C5—C21	176.17 (10)	C26—C21—C22—C23	−0.72 (18)
C3—C4—C5—C21	−5.30 (18)	C5—C21—C22—C23	177.70 (11)
C9—N2—C6—C5	−175.53 (11)	C21—C22—C23—C24	0.0 (2)
C9—N2—C6—C7	2.71 (12)	C22—C23—C24—C25	0.6 (2)
C4—C5—C6—N2	−8.48 (18)	C23—C24—C25—C26	−0.5 (2)
C21—C5—C6—N2	170.23 (10)	C24—C25—C26—C21	−0.30 (18)
C4—C5—C6—C7	173.50 (11)	C22—C21—C26—C25	0.88 (17)
C21—C5—C6—C7	−7.80 (16)	C5—C21—C26—C25	−177.52 (10)
N2—C6—C7—C8	−1.69 (13)	C11—C10—C27—C28	−122.22 (13)
C5—C6—C7—C8	176.58 (11)	C9—C10—C27—C28	58.57 (16)
C6—C7—C8—C9	−0.05 (13)	C11—C10—C27—C32	57.43 (16)
C6—N2—C9—C10	176.68 (11)	C9—C10—C27—C32	−121.78 (13)
C6—N2—C9—C8	−2.75 (12)	C32—C27—C28—C29	−0.18 (18)
C7—C8—C9—N2	1.78 (13)	C10—C27—C28—C29	179.47 (11)
C7—C8—C9—C10	−177.66 (11)	C27—C28—C29—C30	−0.2 (2)
N2—C9—C10—C11	9.00 (19)	C28—C29—C30—C31	0.3 (2)
C8—C9—C10—C11	−171.64 (11)	C29—C30—C31—C32	−0.1 (2)
N2—C9—C10—C27	−171.86 (11)	C30—C31—C32—C27	−0.3 (2)
C8—C9—C10—C27	7.50 (17)	C28—C27—C32—C31	0.4 (2)
C14—N3—C11—C10	174.45 (11)	C10—C27—C32—C31	−179.25 (13)
C14—N3—C11—C12	−2.57 (13)	C16—C15—C33—C34	91.51 (14)
C9—C10—C11—N3	5.97 (19)	C14—C15—C33—C34	−89.12 (14)
C27—C10—C11—N3	−173.17 (11)	C16—C15—C33—C38	−90.45 (15)
C9—C10—C11—C12	−177.67 (12)	C14—C15—C33—C38	88.92 (14)
C27—C10—C11—C12	3.18 (19)	C38—C33—C34—C35	−1.03 (19)
N3—C11—C12—C13	1.97 (14)	C15—C33—C34—C35	177.04 (12)
C10—C11—C12—C13	−174.93 (12)	C33—C34—C35—C36	−0.6 (2)
C11—C12—C13—C14	−0.69 (15)	C34—C35—C36—C37	1.5 (2)
C11—N3—C14—C15	−175.45 (11)	C35—C36—C37—C38	−0.7 (2)
C11—N3—C14—C13	2.16 (13)	C36—C37—C38—C33	−0.9 (2)
C12—C13—C14—N3	−0.87 (14)	C34—C33—C38—C37	1.8 (2)
C12—C13—C14—C15	176.76 (12)	C15—C33—C38—C37	−176.26 (12)
N3—C14—C15—C16	−7.9 (2)	C19—C20—C39—C40	−110.08 (13)
C13—C14—C15—C16	174.91 (12)	C1—C20—C39—C40	71.89 (14)
N3—C14—C15—C33	172.75 (11)	C19—C20—C39—C44	71.77 (15)
C13—C14—C15—C33	−4.41 (17)	C1—C20—C39—C44	−106.26 (13)
C19—N4—C16—C15	−169.25 (11)	C44—C39—C40—C41	0.02 (18)
C19—N4—C16—C17	9.77 (12)	C20—C39—C40—C41	−178.16 (11)
C14—C15—C16—N4	−4.65 (19)	C39—C40—C41—C42	0.2 (2)
C33—C15—C16—N4	174.64 (10)	C40—C41—C42—C43	−0.4 (2)
C14—C15—C16—C17	176.41 (11)	C41—C42—C43—C44	0.3 (2)
C33—C15—C16—C17	−4.31 (16)	C42—C43—C44—C39	0.0 (2)
N4—C16—C17—O1	103.05 (10)	C40—C39—C44—C43	−0.12 (19)
C15—C16—C17—O1	−77.88 (13)	C20—C39—C44—C43	178.09 (12)
N4—C16—C17—C18	−16.00 (12)	C49—N5—C45—C46	−0.4 (2)
C15—C16—C17—C18	163.07 (10)	N5—C45—C46—C47	−0.6 (2)
O1—C17—C18—O2	26.65 (13)	C51—N6—C47—C46	175.09 (14)
C16—C17—C18—O2	142.76 (9)	C50—N6—C47—C46	−8.1 (2)
O1—C17—C18—C19	−101.33 (10)	C51—N6—C47—C48	−3.3 (2)
C16—C17—C18—C19	14.78 (10)	C50—N6—C47—C48	173.57 (13)
C16—N4—C19—C20	176.25 (10)	C45—C46—C47—N6	−176.90 (14)
C16—N4—C19—C18	1.12 (12)	C45—C46—C47—C48	1.6 (2)
O2—C18—C19—N4	−137.49 (10)	N6—C47—C48—C49	176.77 (13)
C17—C18—C19—N4	−10.83 (12)	C46—C47—C48—C49	−1.7 (2)
O2—C18—C19—C20	47.23 (15)	C45—N5—C49—C48	0.2 (2)
C17—C18—C19—C20	173.89 (10)	C47—C48—C49—N5	0.9 (2)
N4—C19—C20—C1	6.04 (19)		

### cis-7,8-Dihydroxy-5,10,15,20-tetraphenylchlorin dimethylaminopyridine monosolvate (2PhH2) Hydrogen-bond geometry (Å, º)

**Table:**

*D*—H···*A*	*D*—H	H···*A*	*D*···*A*	*D*—H···*A*
O1—H1*O*···N5	0.973 (17)	1.727 (17)	2.6968 (14)	174.1 (14)
O2—H2*O*···O1^i^	0.927 (17)	1.882 (17)	2.7798 (12)	162.5 (14)
N1—H1*N*···N2	0.925 (15)	2.346 (15)	2.9064 (13)	118.7 (11)
N1—H1*N*···N4	0.925 (15)	2.383 (15)	2.9518 (13)	119.6 (11)
N3—H3*N*···N2	0.915 (16)	2.292 (16)	2.8868 (13)	122.3 (12)
N3—H3*N*···N4	0.915 (16)	2.458 (15)	2.9766 (14)	116.1 (12)
C37—H37···O2^ii^	0.95	2.51	3.3840 (16)	153
C38—H38···C48^ii^	0.95	2.77	3.6779 (19)	161
C50—H50*B*···N4^ii^	0.98	2.57	3.544 (2)	171

Symmetry codes: (i) −*x*+1, −*y*+1, −*z*+1; (ii) −*x*+2, −*y*+1, −*z*+1.

### [cis-7,8-Dihydroxy-5,10,15,20-tetraphenylchlorinato(2-)]zinc(II)–ethylenediamine–methanol (1/1/0.136) (2PhZn) Crystal data

**Table:**

[Zn(C_44_H_30_N_4_O_2_)]·C_2_H_8_N_2_·0.136CH_4_O	*F*(000) = 1618
*M**_r_* = 776.57	*D*_x_ = 1.397 Mg m^−^^3^
Monoclinic, *P*2_1_/*c*	Cu *K*α radiation, λ = 1.54178 Å
*a* = 10.1249 (3) Å	Cell parameters from 9950 reflections
*b* = 13.5400 (4) Å	θ = 3.3–79.4°
*c* = 27.0447 (8) Å	µ = 1.32 mm^−^^1^
β = 95.1464 (11)°	*T* = 150 K
*V* = 3692.64 (19) Å^3^	Block, black
*Z* = 4	0.27 × 0.25 × 0.18 mm

### [cis-7,8-Dihydroxy-5,10,15,20-tetraphenylchlorinato(2-)]zinc(II)–ethylenediamine–methanol (1/1/0.136) (2PhZn) Data collection

**Table:**

Bruker AXS D8 Quest diffractometer with PhotonIII-C14 charge-integrating and photon counting pixel array detector	7551 independent reflections
Radiation source: I-mu-S microsource X-ray tube	7037 reflections with *I* > 2σ(*I*)
Laterally graded multilayer (Goebel) mirror monochromator	*R*_int_ = 0.024
Detector resolution: 7.4074 pixels mm^-1^	θ_max_ = 79.5°, θ_min_ = 3.3°
ω and phi scans	*h* = −12→11
Absorption correction: multi-scan (SADABS; Krause *et al.*, 2015)	*k* = −16→15
*T*_min_ = 0.606, *T*_max_ = 0.754	*l* = −29→34
21319 measured reflections	

### [cis-7,8-Dihydroxy-5,10,15,20-tetraphenylchlorinato(2-)]zinc(II)–ethylenediamine–methanol (1/1/0.136) (2PhZn) Refinement

**Table:**

Refinement on *F*^2^	Primary atom site location: structure-invariant direct methods
Least-squares matrix: full	Secondary atom site location: difference Fourier map
*R*[*F*^2^ > 2σ(*F*^2^)] = 0.031	Hydrogen site location: mixed
*wR*(*F*^2^) = 0.088	H atoms treated by a mixture of independent and constrained refinement
*S* = 1.04	*w* = 1/[σ^2^(*F*_o_^2^) + (0.0454*P*)^2^ + 1.8191*P*] where *P* = (*F*_o_^2^ + 2*F*_c_^2^)/3
7551 reflections	(Δ/σ)_max_ = 0.001
549 parameters	Δρ_max_ = 0.31 e Å^−^^3^
17 restraints	Δρ_min_ = −0.43 e Å^−^^3^

### [cis-7,8-Dihydroxy-5,10,15,20-tetraphenylchlorinato(2-)]zinc(II)–ethylenediamine–methanol (1/1/0.136) (2PhZn) Special details

**Table:**

Geometry. All esds (except the esd in the dihedral angle between two l.s. planes) are estimated using the full covariance matrix. The cell esds are taken into account individually in the estimation of esds in distances, angles and torsion angles; correlations between esds in cell parameters are only used when they are defined by crystal symmetry. An approximate (isotropic) treatment of cell esds is used for estimating esds involving l.s. planes.
Refinement. The not metal coordinated amino group of an ethylene diamine ligand was refined as disordered. The C-N bonds were restrained to be similar in length. Amine H atom positions were refined and N-H distances were restrained to 0.88 (2) Angstrom. Equivalent H···H and C···H distances were restrained to be similar to each other. Subject to these conditions the occupancy ratio refined to 0.882 (12) to 0.118 (12). A partially occupied methanol molecule is located nearby the major disordered amino group and H-bonded to it. The hydroxyl H atom was restrained to hydrogen bond to a porphyrin N atom of a neighboring complex. Subject to these conditions the occupancy rate refined to 0.136 (4).

### [cis-7,8-Dihydroxy-5,10,15,20-tetraphenylchlorinato(2-)]zinc(II)–ethylenediamine–methanol (1/1/0.136) (2PhZn) Fractional atomic coordinates and isotropic or equivalent isotropic displacement parameters (Å^2^)

**Table:**

	*x*	*y*	*z*	*U*_iso_*/*U*_eq_	Occ. (<1)
Zn1	0.70722 (2)	0.44915 (2)	0.36168 (2)	0.01832 (7)	
O1	0.62030 (12)	0.47166 (9)	0.54271 (4)	0.0308 (2)	
H1	0.6482 (10)	0.4528 (15)	0.5775 (9)	0.046*	
O2	0.56022 (12)	0.65470 (9)	0.51056 (4)	0.0328 (3)	
H2A	0.4922 (17)	0.6092 (15)	0.4949 (8)	0.049*	
N1	0.73694 (12)	0.51455 (9)	0.43385 (4)	0.0204 (2)	
N2	0.68821 (12)	0.58909 (9)	0.33244 (4)	0.0202 (2)	
N3	0.73798 (12)	0.39779 (9)	0.29110 (4)	0.0192 (2)	
N4	0.80823 (12)	0.32619 (9)	0.39012 (4)	0.0201 (2)	
C1	0.76495 (14)	0.46245 (11)	0.47647 (5)	0.0211 (3)	
C2	0.72768 (15)	0.51995 (12)	0.52170 (5)	0.0247 (3)	
H2	0.805841	0.525874	0.546886	0.030*	
C3	0.68812 (16)	0.62230 (12)	0.49999 (5)	0.0256 (3)	
H3	0.755257	0.671994	0.513274	0.031*	
C4	0.70045 (14)	0.60845 (11)	0.44448 (5)	0.0217 (3)	
C5	0.67805 (14)	0.68419 (11)	0.41046 (5)	0.0221 (3)	
C6	0.67411 (14)	0.67471 (11)	0.35816 (5)	0.0214 (3)	
C7	0.65058 (16)	0.75574 (11)	0.32390 (6)	0.0273 (3)	
H7	0.638690	0.823086	0.332289	0.033*	
C8	0.64860 (16)	0.71772 (11)	0.27743 (6)	0.0274 (3)	
H8	0.635401	0.753563	0.247193	0.033*	
C9	0.67021 (14)	0.61307 (11)	0.28244 (5)	0.0216 (3)	
C10	0.66810 (14)	0.54655 (11)	0.24303 (5)	0.0212 (3)	
C11	0.69457 (14)	0.44448 (11)	0.24758 (5)	0.0207 (3)	
C12	0.68755 (15)	0.37502 (12)	0.20741 (5)	0.0255 (3)	
H12	0.658048	0.388023	0.173700	0.031*	
C13	0.73118 (15)	0.28702 (11)	0.22677 (5)	0.0246 (3)	
H13	0.737737	0.226813	0.209116	0.030*	
C14	0.76550 (14)	0.30210 (11)	0.27891 (5)	0.0200 (3)	
C15	0.82584 (14)	0.23173 (11)	0.31236 (5)	0.0205 (3)	
C16	0.85130 (14)	0.24664 (11)	0.36358 (5)	0.0221 (3)	
C17	0.92352 (17)	0.17956 (12)	0.39728 (6)	0.0295 (3)	
H17	0.965688	0.120098	0.388676	0.035*	
C18	0.92010 (17)	0.21707 (13)	0.44358 (6)	0.0304 (3)	
H18	0.958959	0.188554	0.473521	0.036*	
C19	0.84659 (14)	0.30825 (11)	0.43899 (5)	0.0227 (3)	
C20	0.81842 (14)	0.36856 (11)	0.47976 (5)	0.0222 (3)	
C21	0.66512 (16)	0.78725 (11)	0.42976 (5)	0.0257 (3)	
C22	0.54419 (18)	0.83574 (13)	0.42621 (7)	0.0343 (4)	
H22	0.466488	0.801861	0.413263	0.041*	
C23	0.5353 (2)	0.93293 (15)	0.44131 (8)	0.0464 (5)	
H23	0.451780	0.965418	0.438633	0.056*	
C24	0.6477 (2)	0.98287 (14)	0.46029 (8)	0.0492 (5)	
H24	0.641627	1.049810	0.470365	0.059*	
C25	0.7680 (2)	0.93559 (14)	0.46454 (8)	0.0478 (5)	
H25	0.845182	0.969597	0.477894	0.057*	
C26	0.77712 (19)	0.83764 (13)	0.44927 (7)	0.0370 (4)	
H26	0.860658	0.805211	0.452263	0.044*	
C27	0.63996 (15)	0.58672 (11)	0.19152 (5)	0.0222 (3)	
C28	0.51396 (15)	0.62173 (12)	0.17514 (6)	0.0271 (3)	
H28	0.445274	0.620404	0.196869	0.033*	
C29	0.48819 (17)	0.65857 (12)	0.12724 (6)	0.0303 (3)	
H29	0.401410	0.680622	0.116268	0.036*	
C30	0.58747 (18)	0.66344 (12)	0.09542 (6)	0.0317 (3)	
H30	0.569838	0.689923	0.062973	0.038*	
C31	0.71357 (18)	0.62906 (15)	0.11152 (6)	0.0376 (4)	
H31	0.782543	0.632158	0.089955	0.045*	
C32	0.73909 (16)	0.59025 (14)	0.15896 (6)	0.0323 (4)	
H32	0.825091	0.565819	0.169340	0.039*	
C33	0.86940 (14)	0.13604 (11)	0.29145 (5)	0.0213 (3)	
C34	0.95353 (15)	0.13496 (11)	0.25326 (5)	0.0233 (3)	
H34	0.982623	0.195715	0.240408	0.028*	
C35	0.99553 (16)	0.04640 (12)	0.23368 (6)	0.0274 (3)	
H35	1.052341	0.047091	0.207570	0.033*	
C36	0.95451 (17)	−0.04261 (12)	0.25227 (7)	0.0313 (3)	
H36	0.982821	−0.103132	0.238932	0.038*	
C37	0.87209 (17)	−0.04298 (12)	0.29037 (7)	0.0309 (3)	
H37	0.844492	−0.104022	0.303371	0.037*	
C38	0.82924 (16)	0.04546 (11)	0.30982 (6)	0.0263 (3)	
H38	0.772140	0.044189	0.335840	0.032*	
C39	0.85297 (15)	0.32409 (12)	0.53006 (5)	0.0237 (3)	
C40	0.78919 (17)	0.23834 (13)	0.54385 (6)	0.0313 (3)	
H40	0.720053	0.210821	0.522050	0.038*	
C41	0.8254 (2)	0.19268 (15)	0.58903 (7)	0.0394 (4)	
H41	0.782257	0.133703	0.597723	0.047*	
C42	0.92487 (19)	0.23337 (16)	0.62148 (6)	0.0419 (5)	
H42	0.949622	0.202435	0.652456	0.050*	
C43	0.98729 (17)	0.31836 (16)	0.60868 (6)	0.0381 (4)	
H43	1.054483	0.346547	0.631109	0.046*	
C44	0.95294 (15)	0.36380 (13)	0.56300 (6)	0.0291 (3)	
H44	0.997849	0.422012	0.554307	0.035*	
N5	0.50664 (13)	0.40837 (10)	0.36913 (5)	0.0271 (3)	
H5A	0.480 (2)	0.4337 (14)	0.3967 (7)	0.041*	
H5B	0.509 (2)	0.3447 (11)	0.3761 (8)	0.041*	
C45	0.40746 (17)	0.42549 (15)	0.32609 (7)	0.0385 (4)	
H45A	0.329199	0.383047	0.329539	0.046*	
H45B	0.446349	0.405890	0.295254	0.046*	
C46	0.36344 (16)	0.53096 (14)	0.32166 (6)	0.0326 (4)	0.882 (12)
H46A	0.442264	0.574411	0.322569	0.039*	0.882 (12)
H46B	0.311072	0.540812	0.289330	0.039*	0.882 (12)
N6	0.2830 (4)	0.5585 (2)	0.36180 (8)	0.0361 (8)	0.882 (12)
H6A	0.2073 (19)	0.5235 (18)	0.3589 (9)	0.054*	0.882 (12)
H6B	0.257 (3)	0.6185 (13)	0.3606 (9)	0.054*	0.882 (12)
C46B	0.36344 (16)	0.53096 (14)	0.32166 (6)	0.0326 (4)	0.118 (12)
H46C	0.428985	0.567714	0.303754	0.039*	0.118 (12)
H46D	0.277978	0.532993	0.300800	0.039*	0.118 (12)
N6B	0.346 (3)	0.5839 (15)	0.3685 (6)	0.046 (5)	0.118 (12)
H6C	0.305 (16)	0.640 (6)	0.3622 (17)	0.068*	0.118 (12)
H6D	0.425 (5)	0.604 (12)	0.382 (4)	0.068*	0.118 (12)
O3	0.0708 (14)	0.4152 (11)	0.3732 (6)	0.070 (4)	0.136 (4)
H3O	−0.010618	0.402558	0.371916	0.084*	0.136 (4)
C47	0.1402 (18)	0.3301 (15)	0.3759 (7)	0.060 (5)	0.136 (4)
H47A	0.117547	0.291039	0.345850	0.072*	0.136 (4)
H47B	0.118247	0.292480	0.405043	0.072*	0.136 (4)
H47C	0.235366	0.344884	0.378874	0.072*	0.136 (4)

### [cis-7,8-Dihydroxy-5,10,15,20-tetraphenylchlorinato(2-)]zinc(II)–ethylenediamine–methanol (1/1/0.136) (2PhZn) Atomic displacement parameters (Å^2^)

**Table:**

	*U*^11^	*U*^22^	*U*^33^	*U*^12^	*U*^13^	*U*^23^
Zn1	0.02239 (11)	0.01794 (11)	0.01494 (10)	0.00134 (6)	0.00332 (7)	−0.00039 (6)
O1	0.0343 (6)	0.0355 (6)	0.0240 (6)	0.0011 (5)	0.0097 (4)	0.0021 (5)
O2	0.0390 (6)	0.0310 (6)	0.0299 (6)	0.0063 (5)	0.0107 (5)	−0.0054 (5)
N1	0.0240 (6)	0.0202 (6)	0.0175 (6)	0.0000 (4)	0.0033 (4)	−0.0014 (4)
N2	0.0242 (6)	0.0190 (6)	0.0179 (6)	0.0007 (4)	0.0041 (4)	−0.0005 (4)
N3	0.0241 (6)	0.0187 (6)	0.0153 (5)	0.0017 (4)	0.0038 (4)	0.0000 (4)
N4	0.0235 (6)	0.0220 (6)	0.0148 (5)	0.0035 (4)	0.0027 (4)	−0.0006 (4)
C1	0.0224 (7)	0.0249 (7)	0.0161 (6)	−0.0013 (5)	0.0029 (5)	−0.0024 (5)
C2	0.0277 (7)	0.0281 (8)	0.0182 (7)	0.0010 (6)	0.0019 (5)	−0.0036 (6)
C3	0.0332 (8)	0.0245 (7)	0.0191 (7)	−0.0008 (6)	0.0021 (6)	−0.0044 (6)
C4	0.0229 (6)	0.0229 (7)	0.0196 (7)	−0.0010 (5)	0.0032 (5)	−0.0048 (5)
C5	0.0238 (7)	0.0202 (7)	0.0226 (7)	0.0005 (5)	0.0040 (5)	−0.0036 (5)
C6	0.0230 (7)	0.0184 (7)	0.0233 (7)	−0.0002 (5)	0.0044 (5)	−0.0012 (5)
C7	0.0372 (8)	0.0170 (7)	0.0282 (8)	0.0009 (6)	0.0059 (6)	0.0012 (6)
C8	0.0368 (8)	0.0211 (7)	0.0249 (7)	0.0012 (6)	0.0055 (6)	0.0042 (6)
C9	0.0251 (7)	0.0203 (7)	0.0198 (7)	0.0012 (5)	0.0045 (5)	0.0031 (5)
C10	0.0229 (7)	0.0230 (7)	0.0182 (7)	0.0014 (5)	0.0038 (5)	0.0024 (5)
C11	0.0239 (7)	0.0220 (7)	0.0164 (6)	0.0013 (5)	0.0027 (5)	0.0008 (5)
C12	0.0329 (8)	0.0270 (8)	0.0163 (6)	0.0017 (6)	0.0003 (5)	−0.0013 (6)
C13	0.0322 (8)	0.0224 (7)	0.0190 (7)	0.0008 (6)	0.0008 (6)	−0.0042 (5)
C14	0.0229 (6)	0.0203 (7)	0.0172 (6)	−0.0002 (5)	0.0044 (5)	−0.0020 (5)
C15	0.0223 (6)	0.0203 (7)	0.0193 (7)	0.0012 (5)	0.0040 (5)	−0.0015 (5)
C16	0.0244 (7)	0.0225 (7)	0.0198 (7)	0.0047 (5)	0.0038 (5)	−0.0003 (5)
C17	0.0366 (8)	0.0292 (8)	0.0223 (7)	0.0136 (6)	0.0008 (6)	−0.0007 (6)
C18	0.0377 (8)	0.0332 (9)	0.0197 (7)	0.0148 (7)	−0.0009 (6)	0.0018 (6)
C19	0.0254 (7)	0.0247 (7)	0.0179 (7)	0.0034 (6)	0.0015 (5)	0.0000 (5)
C20	0.0241 (7)	0.0262 (7)	0.0162 (6)	0.0008 (5)	0.0021 (5)	−0.0002 (5)
C21	0.0355 (8)	0.0209 (7)	0.0210 (7)	0.0014 (6)	0.0048 (6)	−0.0030 (5)
C22	0.0375 (9)	0.0282 (9)	0.0377 (9)	0.0053 (7)	0.0059 (7)	−0.0034 (7)
C23	0.0566 (12)	0.0319 (10)	0.0513 (12)	0.0176 (9)	0.0074 (9)	−0.0057 (8)
C24	0.0779 (15)	0.0224 (9)	0.0467 (11)	0.0083 (9)	0.0025 (10)	−0.0114 (8)
C25	0.0609 (13)	0.0278 (9)	0.0529 (12)	−0.0050 (8)	−0.0042 (10)	−0.0136 (8)
C26	0.0414 (9)	0.0290 (9)	0.0397 (10)	0.0009 (7)	−0.0015 (7)	−0.0101 (7)
C27	0.0290 (7)	0.0188 (7)	0.0188 (7)	0.0006 (5)	0.0023 (5)	0.0021 (5)
C28	0.0280 (7)	0.0274 (8)	0.0261 (7)	0.0034 (6)	0.0027 (6)	0.0002 (6)
C29	0.0342 (8)	0.0270 (8)	0.0286 (8)	0.0055 (6)	−0.0041 (6)	0.0016 (6)
C30	0.0430 (9)	0.0290 (8)	0.0219 (7)	−0.0030 (7)	−0.0037 (6)	0.0071 (6)
C31	0.0352 (9)	0.0543 (11)	0.0239 (8)	−0.0044 (8)	0.0059 (6)	0.0111 (7)
C32	0.0269 (8)	0.0463 (10)	0.0240 (8)	0.0030 (7)	0.0039 (6)	0.0084 (7)
C33	0.0238 (7)	0.0219 (7)	0.0179 (6)	0.0036 (5)	−0.0002 (5)	−0.0017 (5)
C34	0.0266 (7)	0.0226 (7)	0.0207 (7)	0.0018 (5)	0.0032 (5)	−0.0014 (5)
C35	0.0271 (7)	0.0319 (8)	0.0236 (7)	0.0058 (6)	0.0044 (6)	−0.0044 (6)
C36	0.0329 (8)	0.0237 (8)	0.0369 (9)	0.0081 (6)	0.0015 (7)	−0.0072 (6)
C37	0.0329 (8)	0.0208 (8)	0.0390 (9)	0.0026 (6)	0.0036 (7)	0.0029 (6)
C38	0.0275 (7)	0.0250 (8)	0.0270 (8)	0.0037 (6)	0.0058 (6)	0.0022 (6)
C39	0.0260 (7)	0.0289 (8)	0.0164 (6)	0.0067 (6)	0.0038 (5)	−0.0004 (6)
C40	0.0390 (9)	0.0319 (9)	0.0230 (7)	0.0024 (7)	0.0037 (6)	0.0014 (6)
C41	0.0496 (10)	0.0411 (10)	0.0290 (8)	0.0103 (8)	0.0120 (7)	0.0109 (7)
C42	0.0438 (10)	0.0636 (13)	0.0191 (8)	0.0266 (9)	0.0067 (7)	0.0104 (8)
C43	0.0298 (8)	0.0623 (12)	0.0212 (8)	0.0162 (8)	−0.0031 (6)	−0.0050 (8)
C44	0.0247 (7)	0.0396 (9)	0.0232 (7)	0.0067 (6)	0.0024 (6)	−0.0034 (6)
N5	0.0246 (6)	0.0233 (7)	0.0341 (7)	0.0007 (5)	0.0062 (5)	0.0004 (5)
C45	0.0281 (8)	0.0444 (10)	0.0419 (10)	−0.0002 (7)	−0.0037 (7)	−0.0157 (8)
C46	0.0270 (8)	0.0478 (10)	0.0229 (8)	0.0033 (7)	0.0011 (6)	0.0014 (7)
N6	0.0404 (17)	0.0460 (14)	0.0224 (9)	0.0156 (12)	0.0061 (10)	0.0046 (8)
C46B	0.0270 (8)	0.0478 (10)	0.0229 (8)	0.0033 (7)	0.0011 (6)	0.0014 (7)
N6B	0.035 (12)	0.049 (10)	0.052 (10)	−0.002 (8)	0.002 (8)	−0.006 (7)
O3	0.064 (8)	0.067 (9)	0.078 (10)	−0.017 (7)	−0.003 (7)	0.016 (7)
C47	0.054 (10)	0.072 (12)	0.056 (10)	−0.017 (9)	0.011 (8)	−0.011 (9)

### [cis-7,8-Dihydroxy-5,10,15,20-tetraphenylchlorinato(2-)]zinc(II)–ethylenediamine–methanol (1/1/0.136) (2PhZn) Geometric parameters (Å, º)

**Table:**

Zn1—N2	2.0556 (12)	C25—H25	0.9500
Zn1—N4	2.0660 (12)	C26—H26	0.9500
Zn1—N3	2.0812 (11)	C27—C32	1.394 (2)
Zn1—N5	2.1315 (13)	C27—C28	1.395 (2)
Zn1—N1	2.1399 (12)	C28—C29	1.391 (2)
O1—C2	1.4294 (19)	C28—H28	0.9500
O1—H1	0.99 (2)	C29—C30	1.382 (3)
O2—C3	1.4204 (19)	C29—H29	0.9500
O2—H2A	0.99 (3)	C30—C31	1.392 (3)
N1—C1	1.3593 (19)	C30—H30	0.9500
N1—C4	1.3618 (19)	C31—C32	1.389 (2)
N2—C6	1.3661 (18)	C31—H31	0.9500
N2—C9	1.3865 (18)	C32—H32	0.9500
N3—C14	1.3716 (18)	C33—C34	1.397 (2)
N3—C11	1.3731 (18)	C33—C38	1.397 (2)
N4—C19	1.3654 (18)	C34—C35	1.393 (2)
N4—C16	1.3863 (18)	C34—H34	0.9500
C1—C20	1.382 (2)	C35—C36	1.384 (2)
C1—C2	1.5257 (19)	C35—H35	0.9500
C2—C3	1.544 (2)	C36—C37	1.383 (3)
C2—H2	1.0000	C36—H36	0.9500
C3—C4	1.5292 (19)	C37—C38	1.393 (2)
C3—H3	1.0000	C37—H37	0.9500
C4—C5	1.383 (2)	C38—H38	0.9500
C5—C6	1.417 (2)	C39—C44	1.395 (2)
C5—C21	1.500 (2)	C39—C40	1.395 (2)
C6—C7	1.442 (2)	C40—C41	1.389 (2)
C7—C8	1.356 (2)	C40—H40	0.9500
C7—H7	0.9500	C41—C42	1.389 (3)
C8—C9	1.438 (2)	C41—H41	0.9500
C8—H8	0.9500	C42—C43	1.372 (3)
C9—C10	1.394 (2)	C42—H42	0.9500
C10—C11	1.411 (2)	C43—C44	1.396 (2)
C10—C27	1.4990 (19)	C43—H43	0.9500
C11—C12	1.434 (2)	C44—H44	0.9500
C12—C13	1.359 (2)	N5—C45	1.486 (2)
C12—H12	0.9500	N5—H5A	0.884 (15)
C13—C14	1.4364 (19)	N5—H5B	0.882 (15)
C13—H13	0.9500	C45—C46B	1.498 (3)
C14—C15	1.414 (2)	C45—C46	1.498 (3)
C15—C16	1.401 (2)	C45—H45A	0.9900
C15—C33	1.4960 (19)	C45—H45B	0.9900
C16—C17	1.439 (2)	C46—N6	1.463 (2)
C17—C18	1.354 (2)	C46—H46A	0.9900
C17—H17	0.9500	C46—H46B	0.9900
C18—C19	1.441 (2)	N6—H6A	0.898 (16)
C18—H18	0.9500	N6—H6B	0.853 (16)
C19—C20	1.421 (2)	C46B—N6B	1.479 (14)
C20—C39	1.5001 (19)	C46B—H46C	0.9900
C21—C22	1.385 (2)	C46B—H46D	0.9900
C21—C26	1.387 (2)	N6B—H6C	0.88 (2)
C22—C23	1.383 (3)	N6B—H6D	0.89 (2)
C22—H22	0.9500	O3—C47	1.35 (2)
C23—C24	1.382 (3)	O3—H3O	0.8400
C23—H23	0.9500	C47—H47A	0.9800
C24—C25	1.372 (3)	C47—H47B	0.9800
C24—H24	0.9500	C47—H47C	0.9800
C25—C26	1.394 (3)		
			
N2—Zn1—N4	155.81 (5)	C25—C24—H24	120.1
N2—Zn1—N3	88.38 (5)	C23—C24—H24	120.1
N4—Zn1—N3	87.85 (5)	C24—C25—C26	120.1 (2)
N2—Zn1—N5	102.59 (5)	C24—C25—H25	120.0
N4—Zn1—N5	101.54 (5)	C26—C25—H25	120.0
N3—Zn1—N5	102.84 (5)	C21—C26—C25	120.47 (18)
N2—Zn1—N1	88.29 (5)	C21—C26—H26	119.8
N4—Zn1—N1	88.25 (5)	C25—C26—H26	119.8
N3—Zn1—N1	162.66 (5)	C32—C27—C28	118.52 (14)
N5—Zn1—N1	94.49 (5)	C32—C27—C10	120.79 (13)
C2—O1—H1	109.5	C28—C27—C10	120.69 (13)
C3—O2—H2A	109.5	C29—C28—C27	120.48 (15)
C1—N1—C4	110.22 (12)	C29—C28—H28	119.8
C1—N1—Zn1	124.07 (10)	C27—C28—H28	119.8
C4—N1—Zn1	124.07 (9)	C30—C29—C28	120.72 (15)
C6—N2—C9	106.70 (12)	C30—C29—H29	119.6
C6—N2—Zn1	126.63 (10)	C28—C29—H29	119.6
C9—N2—Zn1	126.20 (10)	C29—C30—C31	119.17 (15)
C14—N3—C11	106.58 (11)	C29—C30—H30	120.4
C14—N3—Zn1	125.90 (9)	C31—C30—H30	120.4
C11—N3—Zn1	124.72 (9)	C32—C31—C30	120.32 (16)
C19—N4—C16	106.72 (12)	C32—C31—H31	119.8
C19—N4—Zn1	126.30 (10)	C30—C31—H31	119.8
C16—N4—Zn1	126.97 (9)	C31—C32—C27	120.76 (15)
N1—C1—C20	125.66 (13)	C31—C32—H32	119.6
N1—C1—C2	111.54 (12)	C27—C32—H32	119.6
C20—C1—C2	122.79 (13)	C34—C33—C38	118.04 (13)
O1—C2—C1	109.69 (12)	C34—C33—C15	120.58 (13)
O1—C2—C3	112.41 (12)	C38—C33—C15	121.37 (13)
C1—C2—C3	103.15 (12)	C35—C34—C33	121.16 (14)
O1—C2—H2	110.5	C35—C34—H34	119.4
C1—C2—H2	110.5	C33—C34—H34	119.4
C3—C2—H2	110.5	C36—C35—C34	119.99 (15)
O2—C3—C4	113.06 (12)	C36—C35—H35	120.0
O2—C3—C2	114.25 (13)	C34—C35—H35	120.0
C4—C3—C2	102.83 (12)	C37—C36—C35	119.65 (14)
O2—C3—H3	108.8	C37—C36—H36	120.2
C4—C3—H3	108.8	C35—C36—H36	120.2
C2—C3—H3	108.8	C36—C37—C38	120.50 (15)
N1—C4—C5	125.69 (13)	C36—C37—H37	119.7
N1—C4—C3	111.67 (12)	C38—C37—H37	119.7
C5—C4—C3	122.63 (13)	C37—C38—C33	120.65 (15)
C4—C5—C6	125.81 (13)	C37—C38—H38	119.7
C4—C5—C21	118.21 (13)	C33—C38—H38	119.7
C6—C5—C21	115.86 (13)	C44—C39—C40	118.47 (14)
N2—C6—C5	126.18 (13)	C44—C39—C20	121.42 (14)
N2—C6—C7	109.70 (12)	C40—C39—C20	120.03 (14)
C5—C6—C7	124.10 (13)	C41—C40—C39	120.89 (17)
C8—C7—C6	107.13 (13)	C41—C40—H40	119.6
C8—C7—H7	126.4	C39—C40—H40	119.6
C6—C7—H7	126.4	C42—C41—C40	119.91 (18)
C7—C8—C9	107.28 (13)	C42—C41—H41	120.0
C7—C8—H8	126.4	C40—C41—H41	120.0
C9—C8—H8	126.4	C43—C42—C41	119.83 (16)
N2—C9—C10	125.87 (13)	C43—C42—H42	120.1
N2—C9—C8	109.15 (13)	C41—C42—H42	120.1
C10—C9—C8	124.93 (13)	C42—C43—C44	120.60 (17)
C9—C10—C11	125.20 (13)	C42—C43—H43	119.7
C9—C10—C27	117.69 (13)	C44—C43—H43	119.7
C11—C10—C27	117.08 (13)	C39—C44—C43	120.28 (17)
N3—C11—C10	124.71 (13)	C39—C44—H44	119.9
N3—C11—C12	109.73 (12)	C43—C44—H44	119.9
C10—C11—C12	125.48 (13)	C45—N5—Zn1	117.93 (11)
C13—C12—C11	106.91 (13)	C45—N5—H5A	111.4 (15)
C13—C12—H12	126.5	Zn1—N5—H5A	110.1 (15)
C11—C12—H12	126.5	C45—N5—H5B	108.8 (14)
C12—C13—C14	107.17 (13)	Zn1—N5—H5B	105.3 (14)
C12—C13—H13	126.4	H5A—N5—H5B	101.8 (18)
C14—C13—H13	126.4	N5—C45—C46B	112.77 (14)
N3—C14—C15	124.61 (12)	N5—C45—C46	112.77 (14)
N3—C14—C13	109.49 (12)	N5—C45—H45A	109.0
C15—C14—C13	125.79 (13)	C46—C45—H45A	109.0
C16—C15—C14	124.48 (13)	N5—C45—H45B	109.0
C16—C15—C33	117.67 (13)	C46—C45—H45B	109.0
C14—C15—C33	117.82 (12)	H45A—C45—H45B	107.8
N4—C16—C15	125.85 (13)	N6—C46—C45	111.44 (17)
N4—C16—C17	109.14 (12)	N6—C46—H46A	109.3
C15—C16—C17	124.99 (13)	C45—C46—H46A	109.3
C18—C17—C16	107.17 (13)	N6—C46—H46B	109.3
C18—C17—H17	126.4	C45—C46—H46B	109.3
C16—C17—H17	126.4	H46A—C46—H46B	108.0
C17—C18—C19	107.31 (13)	C46—N6—H6A	109.2 (15)
C17—C18—H18	126.3	C46—N6—H6B	114.0 (16)
C19—C18—H18	126.3	H6A—N6—H6B	104 (2)
N4—C19—C20	126.10 (13)	N6B—C46B—C45	116.8 (7)
N4—C19—C18	109.61 (13)	N6B—C46B—H46C	108.1
C20—C19—C18	124.29 (13)	C45—C46B—H46C	108.1
C1—C20—C19	125.69 (13)	N6B—C46B—H46D	108.1
C1—C20—C39	119.08 (13)	C45—C46B—H46D	108.1
C19—C20—C39	115.23 (13)	H46C—C46B—H46D	107.3
C22—C21—C26	118.74 (15)	C46B—N6B—H6C	110 (3)
C22—C21—C5	121.41 (15)	C46B—N6B—H6D	109 (3)
C26—C21—C5	119.76 (14)	H6C—N6B—H6D	102 (4)
C23—C22—C21	120.74 (18)	C47—O3—H3O	109.5
C23—C22—H22	119.6	O3—C47—H47A	109.5
C21—C22—H22	119.6	O3—C47—H47B	109.5
C24—C23—C22	120.11 (19)	H47A—C47—H47B	109.5
C24—C23—H23	119.9	O3—C47—H47C	109.5
C22—C23—H23	119.9	H47A—C47—H47C	109.5
C25—C24—C23	119.88 (17)	H47B—C47—H47C	109.5
			
C4—N1—C1—C20	−172.69 (14)	C14—C15—C16—C17	−173.90 (15)
Zn1—N1—C1—C20	21.5 (2)	C33—C15—C16—C17	4.1 (2)
C4—N1—C1—C2	8.29 (16)	N4—C16—C17—C18	1.83 (19)
Zn1—N1—C1—C2	−157.56 (10)	C15—C16—C17—C18	−176.45 (15)
N1—C1—C2—O1	113.02 (14)	C16—C17—C18—C19	−0.5 (2)
C20—C1—C2—O1	−66.03 (18)	C16—N4—C19—C20	−177.20 (14)
N1—C1—C2—C3	−6.94 (16)	Zn1—N4—C19—C20	3.4 (2)
C20—C1—C2—C3	174.00 (14)	C16—N4—C19—C18	2.20 (17)
O1—C2—C3—O2	7.86 (17)	Zn1—N4—C19—C18	−177.24 (11)
C1—C2—C3—O2	125.94 (13)	C17—C18—C19—N4	−1.09 (19)
O1—C2—C3—C4	−115.04 (13)	C17—C18—C19—C20	178.32 (15)
C1—C2—C3—C4	3.04 (15)	N1—C1—C20—C19	−3.6 (2)
C1—N1—C4—C5	173.14 (14)	C2—C1—C20—C19	175.28 (14)
Zn1—N1—C4—C5	−21.0 (2)	N1—C1—C20—C39	175.45 (13)
C1—N1—C4—C3	−6.09 (17)	C2—C1—C20—C39	−5.6 (2)
Zn1—N1—C4—C3	159.75 (10)	N4—C19—C20—C1	−10.3 (2)
O2—C3—C4—N1	−122.20 (14)	C18—C19—C20—C1	170.36 (15)
C2—C3—C4—N1	1.50 (16)	N4—C19—C20—C39	170.55 (14)
O2—C3—C4—C5	58.54 (19)	C18—C19—C20—C39	−8.8 (2)
C2—C3—C4—C5	−177.76 (13)	C4—C5—C21—C22	−108.81 (18)
N1—C4—C5—C6	7.8 (2)	C6—C5—C21—C22	74.97 (19)
C3—C4—C5—C6	−173.08 (14)	C4—C5—C21—C26	74.7 (2)
N1—C4—C5—C21	−168.03 (14)	C6—C5—C21—C26	−101.57 (18)
C3—C4—C5—C21	11.1 (2)	C26—C21—C22—C23	0.7 (3)
C9—N2—C6—C5	176.68 (14)	C5—C21—C22—C23	−175.87 (17)
Zn1—N2—C6—C5	4.2 (2)	C21—C22—C23—C24	−0.1 (3)
C9—N2—C6—C7	−1.77 (16)	C22—C23—C24—C25	−0.6 (3)
Zn1—N2—C6—C7	−174.29 (10)	C23—C24—C25—C26	0.7 (4)
C4—C5—C6—N2	1.8 (2)	C22—C21—C26—C25	−0.6 (3)
C21—C5—C6—N2	177.74 (14)	C5—C21—C26—C25	176.01 (18)
C4—C5—C6—C7	−179.92 (15)	C24—C25—C26—C21	−0.1 (3)
C21—C5—C6—C7	−4.0 (2)	C9—C10—C27—C32	110.01 (18)
N2—C6—C7—C8	0.94 (18)	C11—C10—C27—C32	−68.0 (2)
C5—C6—C7—C8	−177.54 (15)	C9—C10—C27—C28	−69.78 (19)
C6—C7—C8—C9	0.26 (18)	C11—C10—C27—C28	112.19 (17)
C6—N2—C9—C10	−175.80 (14)	C32—C27—C28—C29	0.5 (2)
Zn1—N2—C9—C10	−3.2 (2)	C10—C27—C28—C29	−179.74 (14)
C6—N2—C9—C8	1.93 (16)	C27—C28—C29—C30	−1.6 (2)
Zn1—N2—C9—C8	174.49 (10)	C28—C29—C30—C31	1.3 (3)
C7—C8—C9—N2	−1.36 (18)	C29—C30—C31—C32	0.1 (3)
C7—C8—C9—C10	176.39 (15)	C30—C31—C32—C27	−1.2 (3)
N2—C9—C10—C11	−5.4 (2)	C28—C27—C32—C31	0.9 (3)
C8—C9—C10—C11	177.25 (15)	C10—C27—C32—C31	−178.87 (16)
N2—C9—C10—C27	176.78 (13)	C16—C15—C33—C34	−123.99 (15)
C8—C9—C10—C27	−0.6 (2)	C14—C15—C33—C34	54.12 (19)
C14—N3—C11—C10	−173.52 (14)	C16—C15—C33—C38	55.03 (19)
Zn1—N3—C11—C10	24.5 (2)	C14—C15—C33—C38	−126.86 (15)
C14—N3—C11—C12	3.29 (16)	C38—C33—C34—C35	0.5 (2)
Zn1—N3—C11—C12	−158.64 (10)	C15—C33—C34—C35	179.57 (14)
C9—C10—C11—N3	−6.2 (2)	C33—C34—C35—C36	−0.4 (2)
C27—C10—C11—N3	171.62 (13)	C34—C35—C36—C37	−0.2 (3)
C9—C10—C11—C12	177.44 (15)	C35—C36—C37—C38	0.6 (3)
C27—C10—C11—C12	−4.7 (2)	C36—C37—C38—C33	−0.4 (3)
N3—C11—C12—C13	−1.97 (17)	C34—C33—C38—C37	−0.1 (2)
C10—C11—C12—C13	174.82 (15)	C15—C33—C38—C37	−179.16 (14)
C11—C12—C13—C14	−0.14 (17)	C1—C20—C39—C44	−65.50 (19)
C11—N3—C14—C15	173.12 (13)	C19—C20—C39—C44	113.68 (16)
Zn1—N3—C14—C15	−25.2 (2)	C1—C20—C39—C40	117.69 (17)
C11—N3—C14—C13	−3.37 (16)	C19—C20—C39—C40	−63.13 (19)
Zn1—N3—C14—C13	158.29 (10)	C44—C39—C40—C41	−0.9 (2)
C12—C13—C14—N3	2.20 (17)	C20—C39—C40—C41	176.05 (15)
C12—C13—C14—C15	−174.24 (14)	C39—C40—C41—C42	1.1 (3)
N3—C14—C15—C16	7.9 (2)	C40—C41—C42—C43	−0.2 (3)
C13—C14—C15—C16	−176.19 (14)	C41—C42—C43—C44	−0.8 (3)
N3—C14—C15—C33	−170.08 (13)	C40—C39—C44—C43	−0.2 (2)
C13—C14—C15—C33	5.8 (2)	C20—C39—C44—C43	−177.06 (14)
C19—N4—C16—C15	175.79 (14)	C42—C43—C44—C39	1.0 (2)
Zn1—N4—C16—C15	−4.8 (2)	Zn1—N5—C45—C46B	−78.92 (16)
C19—N4—C16—C17	−2.47 (17)	Zn1—N5—C45—C46	−78.92 (16)
Zn1—N4—C16—C17	176.96 (11)	N5—C45—C46—N6	−69.5 (3)
C14—C15—C16—N4	8.1 (2)	N5—C45—C46B—N6B	−38.3 (15)
C33—C15—C16—N4	−173.93 (13)		

### [cis-7,8-Dihydroxy-5,10,15,20-tetraphenylchlorinato(2-)]zinc(II)–ethylenediamine–methanol (1/1/0.136) (2PhZn) Hydrogen-bond geometry (Å, º)

**Table:**

*D*—H···*A*	*D*—H	H···*A*	*D*···*A*	*D*—H···*A*
O1—H1···N6^i^	0.99	1.73	2.710 (3)	168
O1—H1···N6*B*^i^	0.99	1.54	2.510 (17)	165
O2—H2*A*···O1^i^	0.99	1.82	2.8056 (18)	171
C2—H2···O3^i^	1.00	2.53	3.460 (14)	155
N5—H5*A*···O1^i^	0.88 (2)	2.38 (2)	3.2442 (18)	166 (2)
C46—H46*A*···N2	0.99	2.49	3.368 (2)	148
N6—H6*A*···O3	0.90 (2)	2.08 (2)	2.932 (14)	159 (3)
C46*B*—H46*C*···N2	0.99	2.68	3.368 (2)	126
O3—H3*O*···N4^ii^	0.84	2.20	2.992 (14)	157

Symmetry codes: (i) −*x*+1, −*y*+1, −*z*+1; (ii) *x*−1, *y*, *z*.
